# Fission Yeast Sirtuin Hst4 Functions in Preserving Genomic Integrity by Regulating Replisome Component Mcl1

**DOI:** 10.1038/s41598-018-26476-4

**Published:** 2018-05-31

**Authors:** Lahiri Konada, Shalini Aricthota, Raghavendra Vadla, Devyani Haldar

**Affiliations:** 1 Centre for DNA Fingerprinting and Diagnostics, Survey Nos. 728, 729, 730 & 734, Opposite Uppal Water Tank, Beside BSNL T E Building, Uppal, Ranga Reddy District, Hyderabad, 500039 India; 2 Graduate Studies, Manipal University, Manipal, India

## Abstract

The *Schizosaccharomyces pombe* sirtuin Hst4, functions in the maintenance of genome stability by regulating histone H3 lysine56 acetylation (H3K56ac) and promoting cell survival during replicative stress. However, its molecular function in DNA damage survival is unclear. Here, we show that *hst4* deficiency in the fission yeast causes S phase delay and DNA synthesis defects. We identified a novel functional link between *hst4* and the replisome component *mcl1* in a suppressor screen aimed to identify genes that could restore the slow growth and Methyl methanesulphonate (MMS) sensitivity phenotypes of the *hst4Δ* mutant. Expression of the replisome component Mcl1 rescues *hst4Δ* phenotypes. Interestingly, *hst4* and *mcl1* show an epistatic interaction and suppression of *hst4Δ* phenotypes by *mcl1* is H3K56 acetylation dependent. Furthermore, Hst4 was found to regulate the expression of *mcl1*. Finally, we show that hSIRT2 depletion results in decreased levels of And-1 (human orthologue of Mcl1), establishing the conservation of this mechanism. Moreover, on induction of replication stress (MMS treatment), Mcl1 levels decrease upon Hst4 down regulation. Our results identify a novel function of Hst4 in regulation of DNA replication that is dependent on H3K56 acetylation. Both SIRT2 and And-1 are deregulated in cancers. Therefore, these findings could be of therapeutic importance in future.

## Introduction

The concerted action of histone acetyltransferases (HATs) and histone deacetylases (HDACs) regulate key DNA metabolic processes such as replication, transcription and repair by modulating the acetylation status of histone and non-histone proteins^1–3^. HDACs are classified into four classes based on their sequence homology^4^. The class III HDACs, also called sirtuins require nicotinamide adenine nucleotide (NAD^+^) as a co-factor for deacetylating their substrates^5,6^. Sirtuins function in several important cellular processes including gene expression, heterochromatin maintenance, genome stability and replicative life span^7–9^.

The *S*. *pombe* sirtuin family comprises three sirtuins, *hst2*, *sir2 and hst4*^10^. Fission yeast lacking either *hst2* or *sir2* do not display phenotypes such as slow growth and sensitivity to DNA damaging agents^11^. However, deletion of *hst4* alone results in characteristic phenotypes such as elongated cell morphology (30%), fragmented DNA and sensitivity to DNA damaging agents like ultra-violet (UV) radiation, methyl methane sulphonate (MMS), hydroxy urea (HU) and camptothecin (CPT)^12,13^. Hst4 deacetylates lysine 56 on histone H3^13^. Originally discovered in *S*. *cerevisiae*, H3K56 acetylation is a histone core domain modification, which peaks during the S phase of the cell cycle and is conserved from yeast to higher eukaryotes^13–17^. Acetylation of the H3K56 residue on newly synthesized histone H3 is essential for its deposition on chromatin, while reduction in H3K56 acetylation leads to genomic instability^18,19^. In addition, induction of H3K56 acetylation upon DNA damage facilitates repair, chromatin reassembly and checkpoint recovery^3,19,20^. In *S*. *pombe*, Hst4 down regulation is accompanied by increased H3K56 acetylation during cell cycle, DNA damage and oxidative stress^13,21^. Hst4 has been shown to interact with Myh1, a protein involved in base excision repair and cell cycle checkpoint clamp 9-1-1 complex^21^. However, the exact molecular mechanism by which Hst4 functions in DNA transactions is not understood.

Mcl1 is the *S*. *pombe* homologue of human And-1 and the budding yeast Ctf4. These proteins are characterized by the presence of WD-repeat domains, which aid in protein-protein interactions. Originally identified in a genetic screen for mutants affecting chromosome transmission fidelity^22^, Ctf4 functions in sister chromatid cohesion. It also couples Mcm2-7 helicase to DNA polymerase alpha (Polα) within the replisome complex and facilitates replication by binding to Mcm10^23,24^. Studies using *Xenopus* egg extracts showed the conserved function of Ctf4 in Polα recruitment during DNA replication and cell cycle progression^25^. Reports on human And-1 also indicate its importance in DNA replication through its involvement in the formation of CDC45-MCM2-7-GINS complex (CMG helicase complex)^26^. And-1 participates in several important cellular processes such as checkpoint activation, sister chromatid cohesion and DNA repair^27^. Studies in fission yeast show that Mcl1 is a multifunctional protein that associates with Polα and is required for genome stability, telomere replication, chromosome segregation and DNA repair^28–31^. Deletion of either *hst4* or *mcl1* show similar phenotypes such as elongated cell morphology and sensitivity to DNA damaging agents^12,13,29^. Additionally, these mutants exhibit elevated chromosome loss^12,32^.

In this study, we identified sirtuin Hst4 as a regulator of Mcl1, a *S*. *pombe* orthologue of Ctf4/And-1. We show that the deletion of *hst4* causes S phase delay and DNA synthesis defects, which are partially suppressed by overexpression of *mcl1*. The sensitivity of *hst4*Δ mutant cells to agents that cause replication stress (MMS, HU), with an exception to CPT, are completely rescued by expression of *mcl1*. Our genetic analysis reveals that *mcl1* and *hst4* function in same genetic pathways to preserve genomic integrity. Further, we discovered that during replicative stress Mcl1 levels are altered via Hst4 to maintain genome integrity. Our results indicate that the role of *hst4* in DNA replication is dependent on H3K56 acetylation. Finally, we demonstrate that the human SIRT2 regulates the levels of human Mcl1 orthologue, And-1, revealing conservation of this sirtuin dependent regulatory mechanism in humans.

## Results

### Deletion of *hst4* causes S phase delay

**Figure 1:**
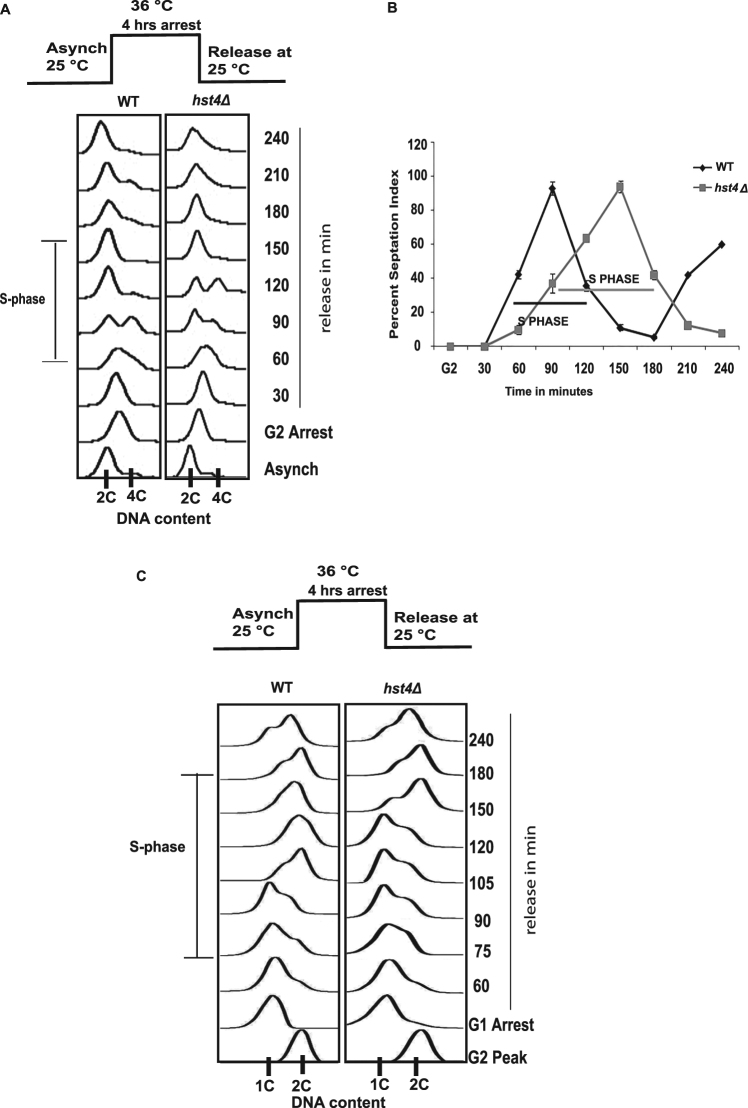
Deletion of *hst4* causes S phase delay. (**A**) Flow cytometry profile showing cell cycle progression of wild type (WT) and *hst4*Δ mutants synchronized at G2/M phase.The *cdc25*-*22* mutant strain (FY4225) and *cdc25*-*22 hst4*Δ (DHP56) were grown in YES medium to log phase at permissive temperature (25 °C) and shifted to restrictive temperature (36 °C) for 4 hr, inducing G2/M arrest. Cells were shifted to permissive temperature (25 °C) after 4 hr, cells were collected every 30 minutes and cell cycle profile was analyzed by flow cytometry. (**B**) Septation index for corresponding cell cycle arrest in (**A**) showing percentage of cells with septa at each time point after release from G2 arrest. Calcofluor was used to stain the septa and DAPI was used to localize the nucleus. (**C**) Flow cytometry profile showing cell cycle progression of wild type (WT) and *hst4*Δ mutants synchronized at G1/S phase. The *cdc10-v50* mutant strain (FY563) and *cdc10-v50 hst4*Δ (DHP91) were grown in YES medium to log phase at permissive temperature (25 °C) and shifted to restrictive temperature (36 °C) for 4 hr, inducing G1/S arrest. Cells were shifted to permissive temperature (25 °C) after 4 hr, cells were collected every 30 minutes and cell cycle profile was analyzed by flow cytometry.

In fission yeast, deficiency of *hst4* results in slow growth and DNA fragmentation phenotypes in the absence of external genotoxic agents^12,13^. Earlier studies have indicated that *S*. *pombe* Hst4 functions in DNA damage response pathways. However, the molecular functions of Hst4 in DNA metabolic pathways are not clear. Cells either arrest or progress slowly through the cell cycle in response to DNA damage^33,34^. To determine the effect of *hst4* deficiency on cell cycle progression, we constructed wild type and *hst4Δ* mutant strains bearing *cdc25*-*22* mutation. We synchronized these wild type and *hst4Δ* mutant strains at G2/M interface using the temperature sensitive *cdc25*-*22* allele. Following their arrest cells were released into cell cycle by lowering the temperature from 36 °C to 25 °C, aliquots of cells were collected at indicated time points and the progression through the cell cycle was monitored using flow cytometry. The results presented in Fig. 1A show that the *hst4*∆ cells display 30 minutes delay in completing S phase compared to the wild type. To further confirm this S phase delay, septation index analysis was carried out which shows synchrony and cell cycle position. Figure 1B shows that wild type cells reached the peak of septation index in 90 minutes after release from arrest, whereas there is a delay in peak of septation index by 1 hour in the *hst4*∆ cells. Next, in order to confirm that the delay of S phase in the *hst4*Δ mutant cells is not due to G2/M delay, we constructed wild type and *hst4Δ* mutant strains bearing *cdc10-v50* mutation. We synchronized these wild type and *hst4*Δ mutant strains at the G1/S phase boundary of cell cycle using the temperature sensitive *cdc10-v50* allele. Following their arrest cells were released into cell cycle by lowering the temperature from 36 °C to 25 °C, aliquots of cells were collected at indicated time points and the progression through cell cycle was monitored using flow cytometry. Figure 1C shows that the wild type cells complete S- phase within 120 minutes after release from the arrest, however, the *hst4Δ* cells progress through the S phase slowly, entering the G2 phase one hour later (180 minutes post-release) than the wild type cells. Altogether, these results suggest that Hst4 is required for progression through the S phase, indicating that it may play a role in DNA replication.

### Deficiency of *hst4* leads to replication defects

**Figure 2:**
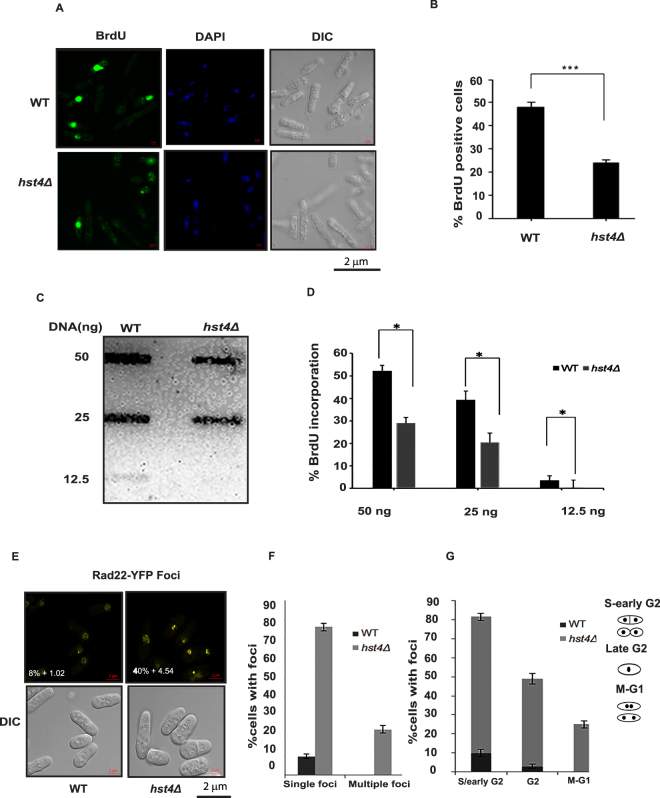
Deficiency of *hst4* leads to replication defects. (**A**) Asynchronously growing wild type (FY4225) and *hst4*Δ (DHP56) strains were grown in presence of BrdU (150 µg/ml) for 30 minutes. Cells were fixed and stained with anti-BrdU antibody and visualized under fluorescent microscope. Bar = 2 µm. (**B**) Quantification of the immunofluorescence data presented in Fig. 2A. The percentage of S-phase cells were determined by counting BrdU positive cells in wild-type and *hst4*Δ mutants. Plotted are the mean values from three independent experiments. Student’s *t* test was used for statistical analysis. *Error bars* indicate mean ± S.D whereas three asterisks represent extremely significant difference, *p*-value < 0.001. (**C**) Slot blot showing BrdU incorporation in genomic DNA of wild type (FY4225) and *hst4*Δ (DHP56) strains using anti-BrdU antibody. Equal amounts of genomic DNA was loaded for wild type and *hst4Δ* mutants. (**D**) Quantification of BrdU incorporation of slot blot analysis. Average and standard deviations from samples of three independent experiments were plotted. Statistical significance *p* < 0.05 between wild-type and *hst4Δ* strains is indicated with a single asterisk. (**E**) Rad22-YFP foci formation by life cell microscopy. Wild-type (ENY0670) and *hst4*Δ mutant (DHP63) containing genomically tagged Rad22-YFP were grown to mid-log phase and percentage of nuclei with at least one Rad22 Foci is shown. Bar = 2 µm. (**F**) Graph showing percent cells with single and multiple Rad22 foci in wild type and *hst4*Δ mutants. Mean values from three independent experiments were used to calculate standard deviation. (**G**) Graph showing percent Rad22 foci according to different cell cycle stages of wild-type and *hst4Δ* mutants. SD was calculated from three independent experiments.

We hypothesized that the delayed S phase progression phenotype of *hst4Δ* mutant cells may be due to replication defects. To examine this possibility, we generated wildtype and *hst4*Δ mutant cells capable of bromodeoxyuridine (BrdU) uptake and incorporation into DNA^35^. The strains were grown for an hour in the presence of BrdU, to label the nascent DNA, and harvested. We determined the BrdU positive cells by immunofluorescence and quantitated them. Figure 2A,B show a two-fold reduction of the BrdU positive population in *hst4* deficient cells compared to wild type cells (47% BrdU positive cells in wild-type versus 23% in *hst4*∆ mutant cells). Further, we quantified the amount of BrdU incorporated into DNA by slot blot, using anti-BrdU antibodies, and observed reduced BrdU incorporation in *hst4*Δ cells (Fig. 2C,D). These results suggest that DNA synthesis is defective in hst4 deficient cells. Many studies directed towards understanding the mechanisms of the S phase delay indicate that problem in replication origin firing and fork progression contribute to reducing the rate of DNA replication^36,37^. The slowing of S phase should result in more number of S phase cells (BrdU positive cells), however, as all S phase cells in the mutant are not actively incorporating BrdU, the number of BrdU incorporating mutant cells decrease. This situation may arise in cases where S phase slowing occurs due to DNA synthesis defects, there are cells which entered S phase but are not actively incorporating BrdU (this situation could be due to replication fork stalling). Although these cells are in S phase, they do not incorporate BrdU. This results in BrdU negative S phase cells. The size of this cell population varies with the severity of DNA synthesis defects. The DNA replication defects generate ssDNA, which could result in DNA damage. Rad22 is a marker for DNA damage as it binds to ssDNA and double strand breaks arising due to replication defects or other exogenous damage^38–40^. Since *hst4*Δ cells are known to accumulate spontaneous DNA damage, we hypothesized that this could be due to replication defects. Therefore, we generated *hst4* deficient strains expressing Rad22-YFP from its genomic loci. A striking increase in Rad22 foci were observed in *hst4*Δ mutants compared to wild type (40% ± 4.54 in *hst4*Δ as compared to 8% ± 1.02 in the wild type, Fig. 2E). We have also observed multiple Rad22 foci appearing only in *hst4*Δ mutants (Fig. 2E,F). Cell cycle position analysis of the Rad22 foci containing cells was also performed to examine whether accumulation of Rad22 foci was occurring in the S phase cells. The S/early G2 phase *hst4*Δ cells exhibited significant accumulation of Rad22 foci (Fig. 2G). Overall, these results confirm that deletion of *hst4* causes defects in DNA replication.

### Overexpression of replisome component Mcl1 suppresses the phenotypes of *hst4* deficient cells

**Figure 3:**
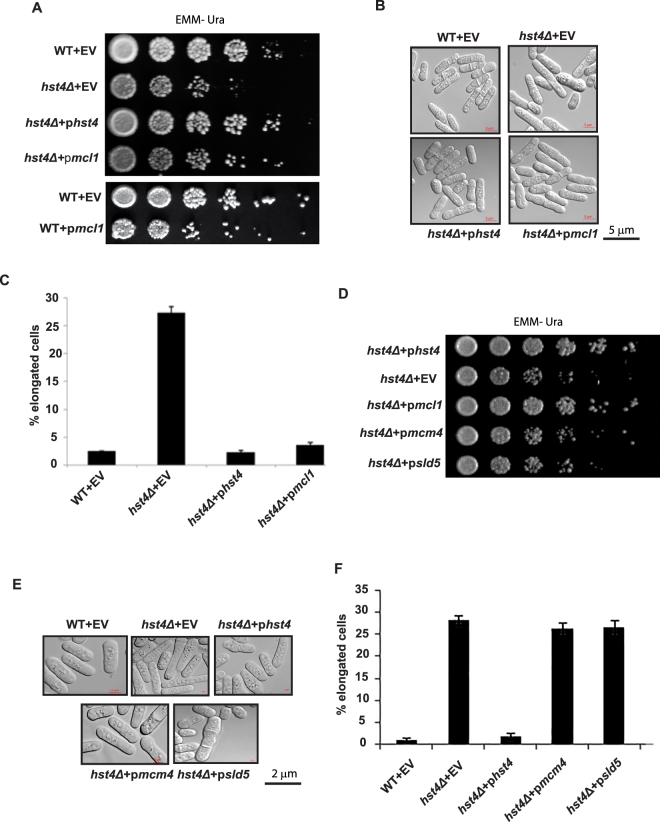
Mcl1 expression rescues the slow growth and elongated morphology phenotypes of *hst4Δ* mutants. (**A**) Wild type strain (ROP191) or *hst4∆* (ROP58) expressing *hst4*, *mcl1* or pRO314 (empty vector) were grown to OD 1, five-fold serially diluted, spotted onto EMM-Ura plates and incubated at 30 °C to assay growth rate. (**B**) Morphology of strains indicated in (**A**) were grown to saturation in liquid EMM medium at 30 °C and observed under phase-contrast microscope. (**C**) Plot showing quantification of elongated cells. Percentage elongated cells were determined by counting at least 100 cells from three independent experiments. Elongated cells were identified as cells that were greater than the size of a normal dividing cell (>14 µm). Error bars represent standard deviation of the mean from three independent experiments. (**D**) *hst4∆* mutant strain (ROP58) expressing *mcm4*, *sld5* or harboring pSLF272 (empty vector) were grown to OD 1, five-fold serially diluted, spotted onto EMM-Ura plates and incubated at 30 °C to assay growth rate. (**E**) *hst4*Δ mutant cells expressing indicated genes were grown in EMM-URA medium at 30 °C and observed under microscope for examining morphology. (**F**) Plot showing quantification of elongated cells in indicated strains. Percentage elongated cells were determined by counting at least 100 cells from three independent experiments. Average and standard deviations were calculated from three independent experiments.

To further understand the pathways functioning aberrantly in the absence of *hst4*, we performed a genetic screen to identify high copy suppressors of the slow growth and DNA damage (MMS) sensitivity phenotypes of *hst4*Δ mutant cells. The *S*. *pombe* genomic library used for this study was generated as described earlier^41^. We have transformed this genomic library in *hst4*∆ mutant cells and selected colonies which were growing like wild type and insensitive to MMS. By isolating plasmids from these cells and sequencing them, we identified multiple classes of genes involved in RNA metabolism, protein transport, and DNA replication as suppressors of *hst4* mutant phenotypes. A clone containing the full length *mcl1* gene was among the suppressors of *hst4Δ* mutant phenotypes. The fission yeast Mcl1, a homologue of *S*. *cerevisiae* Ctf4, is a WD domain containing protein that interacts with many proteins involved in DNA replication and act as a hub for replication factors^32,42^. It also couples the CMG helicase and Polα^43^. To validate Mcl1 as a candidate suppressor of *hst4* mutant phenotypes, *mcl1* gene was cloned into a high copy plasmid (pRO314) and transformed into cells lacking *hst4*. The slow growth phenotype of *hst4*Δ cells was rescued in the presence of *mcl1* as monitored by spot assay (Fig. 3A). As reported previously, expression of *mcl1* alone in the wild type cells leads to slow growth^32^. Thus, the rescue of slow growth of *hst4*Δ cells by *mcl1* expression is specific to its function in *hst4*Δ mutant cells. The elongated cell morphology of cells lacking *hst4* was also rescued by *mcl1* expression and the percentage of elongated cells in *hst4*Δ mutant culture increases in stationary phase. We observed reduction in the percentage of elongated cells (from 28% to 3.5%) in *hst4* deficient cells expressing *mcl1* as compared to *hst4*Δ cells (Fig. 3B,C). In *S*. *cerevisiae*, Ctf4, which is an orthologue of Mcl1, has been shown to couple the CMG helicase complex to Polα. Therefore, we sought to investigate whether *hst4* genetically interact with MCM complex and GINS complex components. We expressed *mcm4* and *sld5* in *hst4Δ* mutants and monitored growth rate by spot assay. Interestingly, expression of *mcm4* and *sld5* had no effect on the slow growth phenotype of *hst4*Δ mutant as compared to *mcl1* (Fig. 3D). The expression levels of Mcl1, Mcm4 and Sld5 in hst4 mutant cells are shown in Supplementary Fig. 1. These results indicate that *hst4* specifically interacts with *mcl1*.

### Mcl1 expression partially rescues the S-phase delay phenotype of *hst4* deficient cells

**Figure 4:**
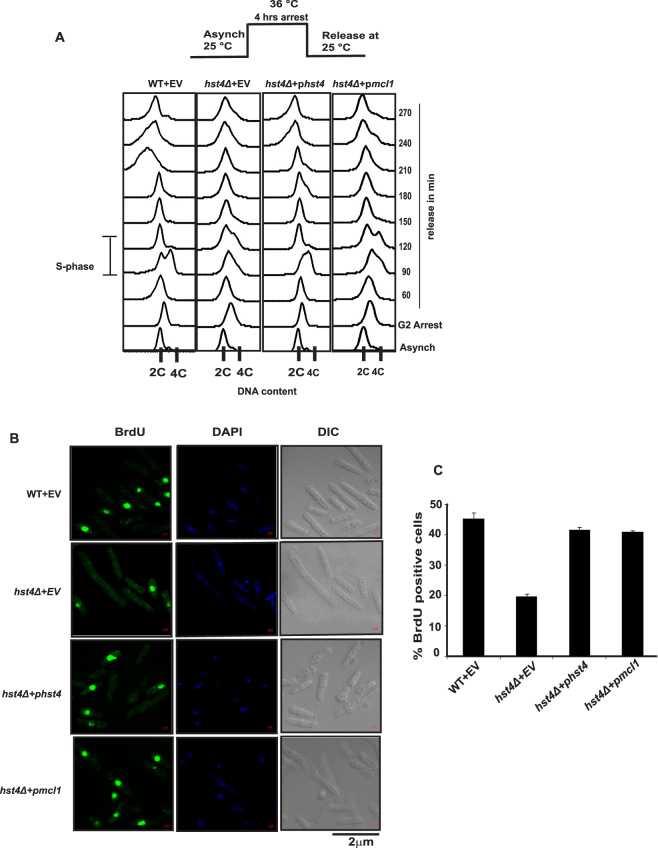
The rescue of S phase delay phenotypes of *hst4Δ* mutants is partly dependent on Mcl1. (**A**) Flow cytometry showing cell cycle progression of wild type (WT) and *hst4*Δ mutants synchronized at G2/M phase. The *cdc25*-*22* mutant strain (FY4225) and *cdc25*-*22 hst4*Δ (DHP56) expressing *hst4*, *mcl1* or pSLF272 (empty vector) were grown in EMM-URA medium to log phase at permissive temperature (25 °C) and shifted to restrictive temperature (36 °C) for 4 hr inducing G2/M arrest, cells were collected every 30 minutes and cell cycle profile was analyzed by flow cytometry. (**B**) Asynchronously growing wild type (FY4225) and *hst4*Δ (DHP56) expressing *hst4*, *mcl1* or pSLF272 (empty vector) were grown in presence of BrdU (150 μg/ml) for 30 minutes. Cells were fixed and stained with anti-BrdU antibody and visualized under fluorescent microscope. Bar = 2 μm. (**C**) Quantification of the immunofluorescence data presented in (B). The percentage of S-phase cells was determined by counting BrdU positive cells. Plotted are the mean values from three independent experiments. *Error bars* indicate mean ± S.D.

The S phase delay and decreased BrdU incorporation observed in cells lacking *hst4* indicate that its deficiency could be resulting in defective DNA replication. Since Ctf4 has been shown to play a crucial role in coupling DNA unwinding and DNA synthesis machineries, we examined whether *mcl1* can rescue the S-phase delay phenotype of the *hst4Δ* mutant cells. Wild type and *hst4Δ* mutant strains were arrested in G2 phase and progression through the cell cycle was monitored using flow cytometry. Our results show that expression of *mcl1* could partially rescue the S-phase delay phenotype of *hst4Δ* deletion mutants (Fig. 4A). However, the rate of S phase progression through S phase was slower in *hst4Δ* cells than the wild type cells (Fig. 4A). To check whether expression of *mcl1* rescues the DNA synthesis defect of *hst4* deletion mutants, *hst4Δ* mutants were grown in the presence of BrdU and the cells which incorporated BrdU were detected by immunofluorescence. The defects in DNA synthesis in *hst4∆* mutants was rescued by expression of *mcl1* (Fig. 4B,C).

### Mcl1 expression rescues *hst4* deficient cells from sensitivity to genotoxic agents

**Figure 5:**
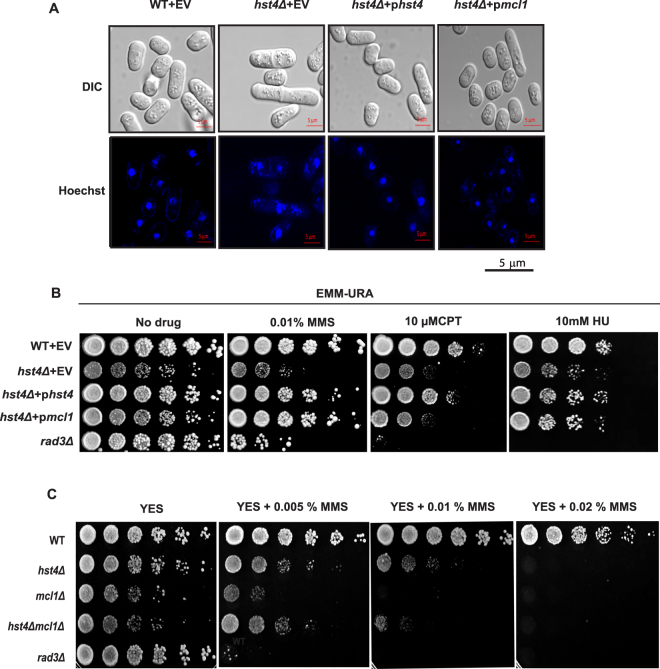
Mcl1 and Hst4 cooperate to promote cell survival following replicative stress. (**A**) Wild type strain (ROP191) or *hst4∆* (ROP58) expressing *hst4*, *mcl1* or pSLF272 (empty vector) were grown to stationary phase, DNA was stained with Hoechst and visualized under confocal microscope. Bar = 2 µm. (**B**) Wild type strain (ROP191) or *hst4∆* (ROP58) or *rad*3*∆* (ROP266) expressing *hst4*, *mcl1* or pSLF272 (empty vector) were five-fold serially diluted and spotted on EMM-Ura plates in the presence or absence of 0.01% MMS or 10 mM HU or 10 µM camptothecin for five days at 30 °C. (**C**) Double deletion mutants of *hst4* and *mcl1* were generated by crossing *hst4∆* (ROP58) and *mcl1∆* (NYSPE19) strains followed by tetrad dissection. Wild type strain (ROP191), *hst4∆* (ROP58), *mcl1∆* (NYSPE19), *hst4∆mcl1∆* (DHP69) and *rad*3*∆* (ROP266) strains were five-fold serially diluted, grown in YES medium in the presence or absence of 0.005, 0.01 and 0.02% MMS and incubated for three days at 30 °C.

To determine whether expression of *mcl1* rescues the DNA fragmentation phenotype of *hst4* deficient cells, we determined nuclear morphology of the *hst4*Δ cells by staining live cells with Hoechst. The results presented in (Fig. 5A) shows that the mutant cells acquired the normal cell and nuclear morphology on expression of *mcl1*. To check whether *mcl1* can suppress MMS sensitivity of *hst4*Δ mutant cells, five-fold serially diluted wild type or *hst4Δ* mutant cells with indicated plasmids were spotted in the presence or absence of MMS on minimal medium. The MMS sensitivity of the *hst4Δ* cells was rescued by expression of *mcl1* (Fig. 5B). The *hst4Δ* mutants are also sensitive to other DNA damaging agents such as camptothecin (CPT) (DNA topoisomerase I inhibitor) and hydroxyurea (HU). To test whether the suppressor *mcl1* can rescue the sensitivity of *hst4Δ* cells to other DNA damaging agents, five-fold serially diluted indicated strains were spotted in the presence or absence of 10 mM HU or 10 µM camptothecin. Mcl1 expression could partially rescued HU sensitivity but not CPT sensitivity of *hst4*∆ mutant cells (Fig. 5B). Overall, these results suggest that Hst4 and Mcl1 function together in cell survival following replicative stress, and these genes specifically interact in certain DNA damage response pathways.

### Mcl1 and Hst4 function in an epistatic manner to maintain genome integrity

To further characterize the genetic interaction between *hst4* and *mcl1*, *hst4*Δ and *mcl1*Δ mutant cells were crossed to generate a double mutant. The results presented in Fig. 5C shows that the double mutants are viable and have phenotypes similar to *hst4*Δ cells, suggesting an epistatic interaction between *hst4* and *mcl1*. However, deletion of *hst4* in *mcl1*Δ cells partially rescues its MMS sensitivity, suggesting a harmful role of *hst4* in the absence of *mcl1*. This effect could be partially due to hyperacetylation of histone H3K56 which may aid in survival and may also be attributed to the function of these proteins in replication and/or cohesion pathways.

### Analysis of H3K56 acetylation on phenotype suppression by Mcl1

**Figure 6:**
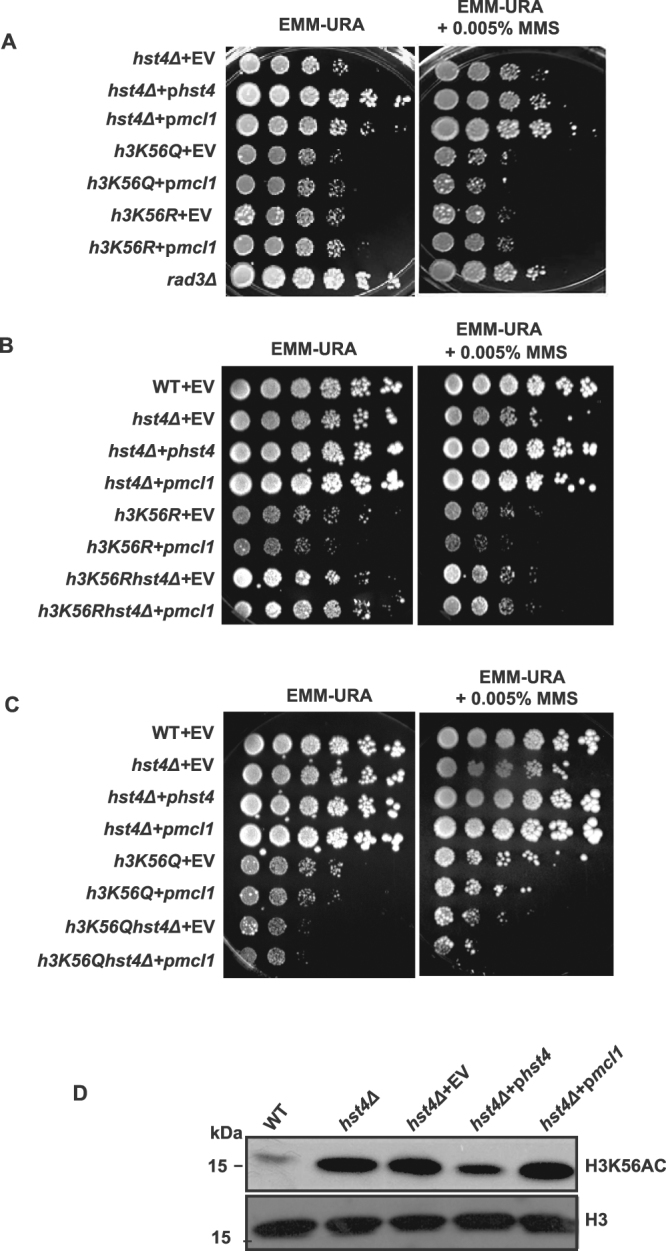
Suppression of *hst4Δ* phenotypes by Mcl1 is dependent of H3K56ac. (**A**) *hst4Δ* (ROP58) expressing *hst4*, *mcl1* or pSLF272 (empty vector), *h3K56Q* (ROP247) or *h3K56R* (ROP253) expressing *mcl1* or pSLF272 (empty vector) and *rad3Δ* (ROP266) were grown to OD 1, five-fold serial dilutions were prepared and spotted on to EMM-Ura or EMM-Ura + 0.005% MMS plates. (**B**) *hst4Δ* (ROP58) expressing *hst4*, *mcl1* or pSLF272 (empty vector), *h3K56R* (ROP253), *h3K56Rhst4Δ* (ROP275) expressing *mcl1* or pSLF272 (empty vector) grown to OD 1, five-fold serial dilutions were prepared and spotted on to EMM-Ura or EMM-Ura + 0.005% MMS, or EMM-Ura + 0.01% MMS plates. (**C**) *hst4Δ* (ROP58) expressing *hst4*, *mcl1* or pSLF272 (empty vector), *h3K56Q* (ROP247), *h3K56Qhst4Δ* (ROP276) expressing *mcl1* or pSLF272 (empty vector) grown to OD 1, five-fold serial dilutions were prepared and spotted on to EMM-Ura or EMM-Ura + 0.005% MMS, or EMM-Ura + 0.01% MMS plates. (**D**) Indicated strains were grown to mid log phase at 30 °C, whole cell extracts were prepared and Western blot was performed using antibodies against H3K56ac and histone H3.

The histone H3 lysine 56 (H3K56) is deacetylated by Hst4. Interestingly, the phenotypes of *H3K56R* and *H3K56Q* mutants, which mimic constitutive deacetylated and acetylated states respectively, are similar to *hst4*Δ cells^13^. Therefore, we examined whether expression of *mcl1* could rescue the slow growth and MMS sensitivity phenotypes of *hst4*Δ cells. The results presented in Fig. 6A show that *mcl1* expression could not suppress the phenotypes of these mutants, indicating H3K56ac may play an important role in this pathway. To further confirm the function of H3K56ac, we have investigated whether expression of *mcl1* can rescue the slow growth and MMS sensitivity phenotypes of *H3K56Qhst4*Δ and *H3K56Rhst4*Δ double mutant cells. The results presented in Fig. 6B show that the slow growth phenotype of *H3K56Rhst4*Δ double mutant cells was partially recovered by *mcl1* expression. However, it could not rescue the MMS sensitivity phenotype. Furthermore, expression of *mcl1* could not rescue the slow growth and MMS sensitivity phenotypes of *H3K56Qhst4*Δ double mutant cells (Fig. 6C). Since the phenotypes of *hst4*Δ mutant cells are mainly attributed to increased H3K56ac levels, we analyzed the status of H3K56ac in *hst4*Δ mutant cells on expression of Mcl1 by immunoblotting. The levels of acetylation in *hst4*Δ mutant cells remain unchanged on expression of *mcl1* indicating *mcl1* functions downstream to H3K56ac (Fig. 6D). In summary, these results suggest that the suppression of *hst4Δ* mutant phenotypes by expression of *mcl1* is dependent on H3K56ac pathway.

### Hst4 regulates the expression of replisome component Mcl1

**Figure 7:**
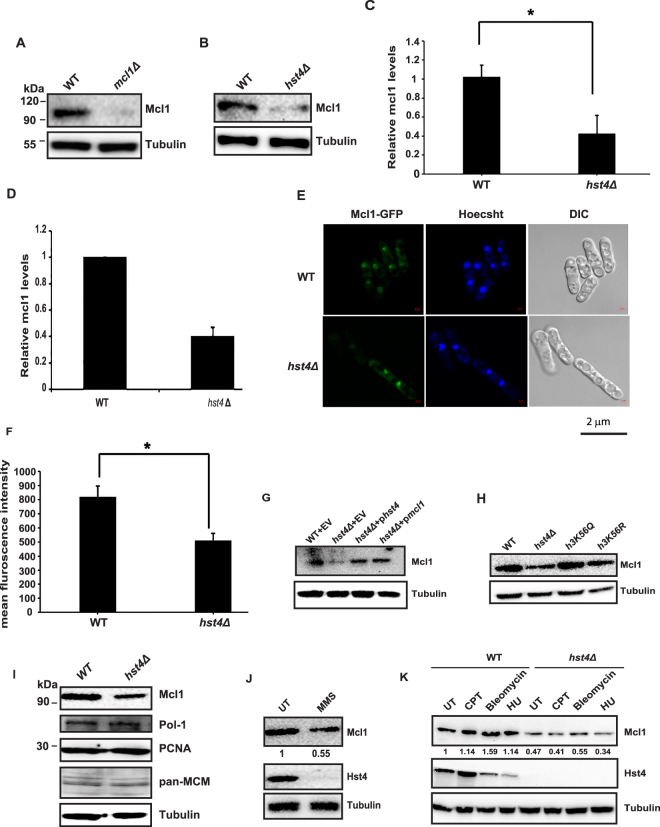
Hst4 regulates mcl1 expression. (**A**,**B**) Wild type (ROP191), *hst4*∆ (ROP58) and *mcl1*∆ (NYSPE19) strains were grown asynchronously to mid log phase in YES medium. Whole cell lysates were prepared and the levels of Mcl1 were monitored by Western blotting using anti-And-1 antibody. (**C**) The Mcl1 levels in WT and *hst4*Δ mutants were quantified relative to tubulin levels by densitometric analysis using Image J software. Error bars: standard deviation of the mean of densitometry values (three independent experiments). Statistical significance *p* < 0.05 between WT and *hst4Δ* strains is indicated with a single asterisk. (**D**) qPCR quantification of mcl1 levels in WT and *hst4*Δ mutants normalized with actin levels. Error bars represent standard deviation. (**E**) Live cells of wild type (YAP50) and *hst4*Δ mutant cells bearing endogenous GFP tagged *mcl1* gene (DHP57) were visualized under confocal microscope. Bar = 2 µm. (**F**) Quantification of nuclear GFP signal in wild type versus *hst4Δ* described in (**D**). The intensity of Mcl1-GFP foci was analyzed using Image J software. Approximately 50 cells from three independent experiments against each genotype is plotted. Statistical significances between WT and *hst4*Δ indicated with single asterisk, *p* < 0.05. (**G**) Cell lysates from wild type strain (ROP191) or *hst4∆* (ROP58) expressing *hst4*, *mcl1* or pSLF272 (empty vector) were prepared and Mcl1 levels were analyzed by Western blotting using anti-And-1 antibody. (**H**) Logarithmically grown cultures of WT (ROP191), *hst4*Δ (ROP58), *h3K56Q* (ROP247) and *h3K56R* (ROP253) strains were lysed and Western blot was performed to check the levels of Mcl1. (**I**) WT(ROP191) and *hst4*∆ (ROP58) strains were grown asynchronously. Whole cell lysates were prepared and the levels of Mcl1, Pol1, PCNA, MCMs were monitored by Western blotting using individual antibodies. (**J**) Cell lysates from WT strains untreated or treated with MMS were analyzed by Western blot using anti-And-1antibody. (**K**) Assessment of Mcl1 and Hst4 levels by Western blot in WT (ROP191) and *hst4*∆ (ROP58) cells untreated or treated with either CPT or bleomycin or HU. For all the Western blots, tubulin was used as loading control. The amount of Mcl1 was quantitated relative to tubulin. The average relative intensity of bands was calculated from three independent experiments.

DNA replication is a tightly regulated process. The coupling between CMG helicase and DNA polymerases is a crucial determinant for DNA replication. In *S*. *cerevisiae*, it has been shown that Mcl1 homologue Ctf4 is a major target of H3K56ac pathway^44^. Our data showed that Mcl1 expression could suppress *hst4*Δ mutant phenotypes. Therefore, we hypothesized that Mcl1 levels might be low in *hst4*Δ mutants resulting in the slow S phase progression. And-1 is the human orthologue of fission yeast Mcl1. The anti-And-1 antibody raised against the conserved C-terminal region of And-1 was used to examine the protein levels of Mcl1. The C-terminal region of And-1 consists of multiple sepB domains, which are highly conserved amongst the members of And-1/Mcl1 family (Supplementary Fig. 2). To verify the specificity of And-1 antibody in yeast, extracts from wild type and *mcl1*Δ strains were prepared and analyzed by immunoblotting using anti-And-1 antibody. The 93 kDa Mcl1 protein was not recognized in *mcl1* deletion mutants confirming the specificity of antibody (Fig. 7A). To examine whether Mcl1 expression is altered in *hst4*Δ mutant cells, we analyzed Mcl1 levels in wild type and *hst4*Δ mutant cells at the RNA and protein level. We observed a two-fold reduction in the protein levels of Mcl1 by Western blot in *hst4*Δ mutant cells as compared to wild type (Fig. 7B,C). We also observed corresponding two-fold reduction inMcl1 transcript levels in *hst4*Δ mutant cells compared to wild type cells, by quantitative RT-PCR (Fig. 7D). Next, the expression of Mcl1 in wild type and *hst4*Δ mutant strains bearing endogenous GFP-tagged *mcl1* gene was analyzed using fluorescence microscopy. Our results confirmed a decrease of Mcl1 expression in *hst4*Δ strains (Fig. 7E,F). To further confirm the regulation of Mcl1 by Hst4, we checked whether overexpression of Hst4 can rescue Mcl1 expression. Overexpression of *hst4* or *mcl1* in *hst4*Δ mutant cells reveal that Hst4 is required for the expression of Mcl1(Fig. 7G). To test whether hyperacetylation of H3K56 in *hst4*Δ mutant cells affect Mcl1 expression, we analyzed *mcl1* levels in *h3K56Q* and *h3K56R* mutants. Mcl1 expression was unaffected in these mutants (Fig. 7H). This result suggests that functional acetyl group at H3K56 might be required for down regulation of Mcl1. In order to check whether deletion of *hst4* affect the expression of other replication proteins, we analyzed the expression of other replication proteins such as Pol1(Polα that binds to Mcl1), MCM complex (helicase component), and PCNA (clamp loader) in *hst4*Δ mutant cells. We did not observe any significant differences in the expression of other replication proteins (Fig. 7I). Collectively, these results reveal that Hst4 specifically regulates Mcl1 transcriptionally.

### Mcl1 expression is reduced upon MMS treatment

It has been reported that expression of Hst4 is down regulated on MMS treatment^13^. To investigate the regulation of Mcl1 by Hst4, the expression of Mcl1 and Hst4 was analyzed in wild type cells treated with different DNA damaging agents, by immunoblotting. Interestingly, Hst4 levels were down regulated on MMS, bleomycin and HU treatment but not on CPT treatment^13^. We observed that the Mcl1 levels were also down regulated on MMS treatment (Fig. 7J). However, when cells were exposed to other DNA damaging agents, we did not observe any significant changes in Mcl1 expression (Fig. 7K).

### Conservation of the regulation of Ctf4/Mcl1/And-1 by sirtuins from yeast to human cells

**Figure 8:**
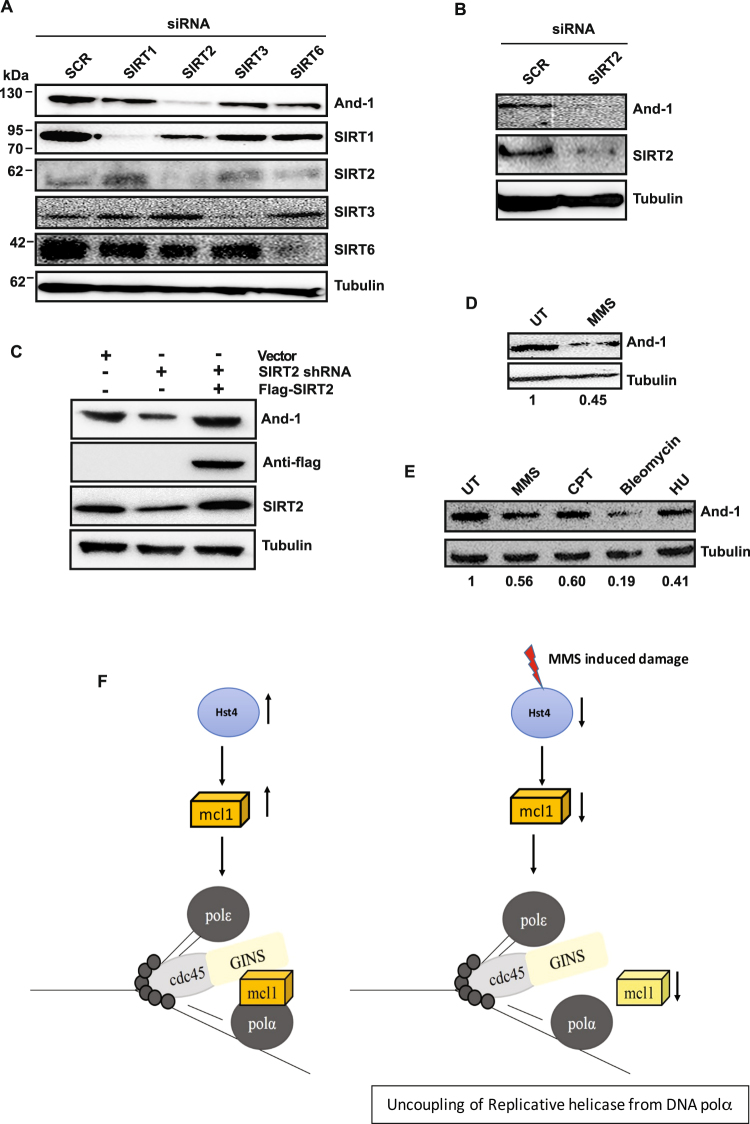
Human Sirtuin2 regulates Mcl1/And-1 expression. (**A**) HeLa cells were transfected with scramble, SIRT1, SIRT2, SIRT3 and SIRT6 siRNA. At 48 h post transfection, whole cell extracts were prepared and And-1 expression was detected by Western blot. The expression of indicated sirtuins was detected by respective antibodies. (**B**) Western blot showing depletion of hSIRT2 reduces And-1 expression. (**C**) HeLa cells were transfected with SIRT2 shRNA targeted to 3′ end of endogenous mRNA for 48 h and then transfected with Flag-SIRT2 for 24 h. Whole cell lysates were analyzed for indicated proteins by Western blot (**D**) Western blot showing And-1 expression in cell lysates from HeLa cells untreated or treated with 0.005% MMS. (**E**) Western blot showing And-1 expression in HeLa cells treated with indicated damaging agents. The amount of And-1 was quantitated relative to tubulin. The average relative intensity of bands is from three independent experiments. (**F**) Model showing how decrease in Mcl1 level mediated by down regulation of Hst4 may cause stalling of replication fork to preserve genomic integrity. In wild-type cells, Mcl1 couples replicative helicase and polymerase leading to efficient DNA synthesis. Under replicative stress such as MMS, Hst4 is down regulated resulting in decrease in level of Mcl1 causing uncoupling and stalling of replication fork to prevent DNA synthesis.

The functions of Mcl1 in replication and sister chromatid cohesion are conserved from budding yeast to vertebrates. We investigated whether the expression of human And-1(Mcl1 orthologue) is regulated by sirtuins in mammals. Mammals have seven sirtuins^12^. Mammalian SIRT1 and SIRT2 are localized in both cytoplasm and nucleus^5,45,46^, SIRT3 is mitochondrial and SIRT6 is a predominant nuclear protein^47,48^. We transfected HeLa cells with siRNA to knockdown SIRT1, SIRT2, SIRT3, and SIRT6 proteins and checked the levels of And-1 by immunoblotting. Depletion of SIRT2 reduced And-1 expression significantly compared to other sirtuins (Fig. 8A,B). To further confirm the regulation of And-1 by SIRT2, we transfected SIRT2 shRNA construct targeted to 3′ end of endogenous mRNA in HeLa cells and overexpressed with FLAG-SIRT2. We observed rescued And-1 expression on overexpression of FLAG-SIRT2 (Fig. 8C). To examine whether human And-1 is down regulated on MMS treatment and on treatment with other DNA damaging agents, cell extracts were prepared from either untreated or treated HeLa cells and analyzed by Western blot using anti-And-1 antibody (Fig. 8D). We observed significant decrease in And-1 level upon MMS, bleomycin, and HU treatments (Fig. 8E). These results indicate that SIRT2 depletion leads to decreased And-1 expression in mammals suggesting that this regulatory mechanism is evolutionarily conserved.

## Discussion

We have identified a novel regulatory role of sirtuin family HDAC Hst4 in the regulation of Mcl1, which acts as a hub for replication proteins^42^. In the absence of *hst4*, cells progress through S phase slowly as their DNA is damaged (Fig. 1). This slowing of S phase could be mediated via activation of intra S phase checkpoint, which provides cells time to repair the damaged DNA before its replication^37,49,50^. Our earlier work has shown synthetic lethal interaction between the mediator of intra S phase checkpoint *cds1* and *hst4*^13^. Many studies directed towards understanding the mechanisms of the S phase delay indicate that replication origin firing and fork progression contribute to reducing the rate of DNA replication. Here, we present data showing reduction of DNA synthesis on deletion of *hst4*, indicating it is required for DNA replication (Fig. 2). Interestingly, in fission yeast, similar phenotype results from reduced levels of a component of replicative MCM helicase Mcm2, which stalls replication fork progression and also cause S phase delay^33,36,37^. Studies towards understanding the cause of S phase delay phenotype in *S*. *pomb*e and other eukaryotes indicate that it could be either defect in origin firing or fork progression or both^33,36,51^. However, not much is known about the role of sirtuins in slowing of DNA replication. In budding yeast, Sir2, a sirtuin family HDAC, has been implicated in negative regulation of DNA replication^52^. On contrary, two other studies on HDACs showed that Rpd3, Sir2, and Hst1 promote replication initiation at many origins^53,54^. In humans, SIRT1 has been shown to function in initiation of DNA replication^55,56^. Although these reports indicate the role of sirtuins in DNA replication, the mechanisms are not very clear. In order to uncover novel functions of Hst4, we performed a high-copy suppressor screen and identified Mcl1 (Fig. 3A), a protein which interacts with Polα and also functions in establishing sister chromatid cohesion. In *Schizosaccharomyces pombe*, *mcl1* was first reported as an essential gene required for maintaining genome stability and deletion of which caused chromosome segregation defects^32^. However, another report showed that cells lacking *mcl1* were viable but displayed sick phenotype^29^. The budding yeast orthologue of Mcl1, Ctf4 is a multifunctional protein involved in maintaining genomic integrity but not an essential gene^22^. Recent reports have shown that Ctf4 is involved in coupling replicative CMG helicase to Polα^43^. Here we show expression of *mcl1* partially suppresses the S- phase delay phenotype observed in *hst4*Δ mutant cells. We report reduced expression of *mcl1* in *hst4* deletion mutants (Fig. 7). Our results have shown for the first time that Hst4 is required for efficient DNA replication. Reduction in the expression of replication proteins affect the replication process^36^. Limited *mcl1* expression may lead to accumulation of ssDNA and uncoupling of lagging and leading strand replication. The presence of increased Rad22 foci in the *hst4Δ* cells especially in the S/early G2 phase (Fig. 2) also indicate the accumulation of high amount of ssDNA. The total levels of Polα and Mcm remained unchanged in *hst4*Δ mutants (Fig. 7H). Budding yeast *CTF4* mutants fail to stabilize the helicase complex thereby resulting in defective DNA synthesis^57^. Human And-1 depletion also leads to replication defects^26,27^. We demonstrated that expression of Mcl1 suppresses the growth defects and restores MMS and HU sensitivity of *hst4*Δ mutants but not CPT sensitivity of *hst4*Δ cells. DNA damage by MMS causes stalling of replication forks and leads to activation of Cds1 dependent replication checkpoint, while CPT traps Topoisomerase I on DNA and leads to collapse of replication forks due to replisome run off^58^. Suppression of sensitivity of *hst4*Δ cells to MMS and HU by Mcl1 suggests that Hst4 may function in replication fork stabilization and recovery. The differential sensitivity to various damaging agents also points towards distinct roles for Hst4 in these repair pathways. We propose that recovery of replication forks after CPT treatment may require Hst4 but is independent of Mcl1 function.

Hst4 deacetylates the histone H3K56 after its acetylation dependent incorporation into chromatin^3,16^. The dynamic regulation of H3K56ac is required for cell survival on replication stress as *H3K56R* or *H3K56Q* mutants, which mimic constitutive deacetylated and acetylated states respectively, are sensitive to DNA damage. The phenotype of cells lacking *hst4* are attributed to hyperacetylated chromatin and are similar to H3K56R and H3K56Q mutants^13^. Here, we showed that expression of *mcl1* could rescue the phenotypes of *hst4Δ* mutants. However, our data showed the sensitivity of these mimics to DNA damaging agents, are not recovered by expression of *mcl1*, indicating functional acetyl group might be required for phenotype suppression. Previous reports have shown that acetyl lysine and glutamine are not perfect mimic due to their structural differences^59^. The expression of *mcl1* does not alter the H3K56ac levels, suggesting that *mcl1* functions downstream of H3K56ac. This study depicts that Mcl1 may also function in H3K56 acetylation pathway as in the case of *S*. *cerevisiae*^44,60,61^. Additionally, work in *S*. *cerevisiae* shows that suppression of *hst3Δ hst4Δ* is brought about by deletion of *ctf4*Δ, which is contrary to our observation in fission yeast where deletion of *hst4* increase survival of *mcl1Δ* cells on replication stress^59^. This difference could be partly explained by the fact that in budding yeast, *ctf4*Δ mutants have milder phenotypes compared to *hst3Δ hst4Δ*, whereas *S*. *pombe mcl1*Δ mutants are more sensitive compared to *hst4*Δ mutants suggesting additional roles played by Mcl1.

The suppression of *hst4Δ* mutant phenotypes by expression of *mcl1* is further correlated to its low cellular levels (Fig. 7). Additionally, Mcl1 is regulated by Hst4 in response to replication stress generated by MMS treatment. In *S*. *pombe*, it has been shown that upon replication stress, cell signals for degradation of replisome components to maintain genomic integrity^62^. Genetic analyses in *S*. *cerevisiae* suggest that in the presence of replicative stress H3K56 acetylation uncouples the Cdc45–Mcm2-7–GINS DNA helicase complex and DNA polymerases through the replisome component Ctf4. However, they could not detect significant decrease in Ctf4 level under replication stress^44^. This pathway is dependent on regulation of H3K56ac by Hst3p and Hst4p and represents a key mechanism for maintenance of genome stability^61^. However, our results have indicated that there could be indeed a direct role of Hst4 in the regulation of Mcl1 at the transcriptional level. Future studies towards understanding the molecular players that are involved in transcriptional regulation of Mcl1, along with Hst4, needs to be carried out. Although currently there is no evidence, it is tempting to speculate how reduction of *mcl1* expression may affect DNA replication process on replicative stress. In *S*. *pombe*, Mcl1 has been shown to interact with Polα. However, whether Mcl1 interacts with Mcm complex and GINS complex has not been studied. We propose that Mcl1 couples replicative helicase to DNA polymerase in *S*. *pombe*, and on replicative stress reduction of Hst4 levels leads to decreased *mcl1* expression (Fig. 7), causing uncoupling of replicative CMG helicase from polymerase to stall DNA synthesis, thus maintaining genomic integrity (Fig. 8F). Sirtuins are conserved in higher eukaryotes. Mammalian sirtuin SIRT2 plays a major role in maintaining genomic integrity^63^. Human SIRT2 deacetylates histone H3 on lysine 56, a signature chromatin mark involved in DNA replication and repair^64^. Our study shows that depletion of SIRT2 leads to reduced And-1 expression, a conserved regulatory mechanism of replication protein regulation by sirtuins. These data points out that SIRT2 might be the functional human homologue of fission yeast Hst4.

We have uncovered the novel Hst4-Mcl1 axis for regulation of DNA replication on replicative stress. Our results indicate that this pathway might be conserved in mammalian cells. The knowledge of such regulatory mechanism involving sirtuins during replicative stress will be useful in designing therapeutics against diseases, such as cancer where sirtuins and And-1 are deregulated.

## Materials and Methods

### Yeast Strains, media and growth conditions

Yeast strains used in this study are listed in Table 1. Standard techniques were used for growth, transformation and genetic manipulations^65^. *S*. *pombe* strains were grown in yeast extract plus supplements (YES) or Edinburgh minimal media (EMM) at 32 °C on plate or in liquid media. Transformations were done using lithium acetate protocol. 10 millilitres of culture were grown to an optical density OD_600_ = 1. The cells were washed with 10 ml of sterile water once followed with 5 ml of Tris-EDTA (TE) plus 0.1 M lithium acetate. Cells were resuspended in 0.1 ml of TE plus 0.1 M lithium acetate and incubated for 1 h on a roller drum at 32 °C. 5 µl of 10 mg/ml carrier DNA (salmon sperm DNA) and 1 µg of plasmid DNA was added to 0.1 mL of cells and incubated at 32 °C for 30 min. Then, 0.7 ml of polyethylene glycol solution (40% polyethylene glycol) was added to the cells and incubated at 32 °C for 1 h. The cells were heat shocked for 5 min at 42 °C, resuspended in 0.2 ml of water and plated on EMM plates supplemented without uracil.

**Table 1:** List of strains used in the study.

Strain	Genotype	Source
FY4225	*h- cdc25*-*22 leu1*::*hENT-leu1* + *his7*-*366*::*hsv-tk-his7* + *ura4? ade6-M*210	Susan. L. Forsburg
DHP56	*h- hst4Δ*::*KanMX6 cdc25*-*22 leu*1::*hENT-leu*1 + *his7*-3*66*::*hsv-tk-his7* + *ura4? ade6-M210*	This work
ROP191	*h- ade6*-*210 arg*3*-D4 his3-D1 leu1*-*32 ura4-D18*	Pillus L
ROP57	*h-ade6*-*216 arg3-D4 his3-D1 leu1*-*32 ura4-D18 hst4Δ*::*his3* +	Pillus L
YAP50	*h* + *leu1*-*32 ura4-D18 his3-D1 mcl1* + *-GFP-kan*	Takashi Toda
DHP57	*h+leu1*-*32 ura4-D18 his3-D1 hst4Δ*::*his3* + *mcl1* + *-GFP-kan*	This work
ROP83	*h-leu1*-*32 ura4-D18 cds1Δ*::*ura4*	Lab collection
ROP266	*h* + *ura4-D18 rad3Δ*::*ura4* + *leu1*-*32 ade6?704/216 arg*+	Lab collection
ROP247	*h? h3*.*2-K56R h3*.*1Δ/h4*.*1Δ*::*his3* + *h3*.*3Δ /h4*.*3Δ*::*arg3* + *leu1*-*32 ura4-D18 his3-D1 arg3-D4 ade6*-210	Lab collection
ROP253	*h?h3*.*2-K56Q h3*.*1Δ /h4*.*1Δ*::*his3* + *h3*.*3Δ /h4*.*3Δ*::*arg3* + *leu1*-*32 ura4-D18 his3-D1 arg3-D4 ade6*-*210*	Lab collection
NY SPE19	*h* + *mcl1Δ*::*ura4* + *ura4-D18 leu1*-*32 his7*-*366 ade6-M210*	Yashuhiro Tsutsui
DHP59	*h- hst4Δ*::*his3* + *mcl1Δ*::*ura4* + *ade6*-*216 arg3-D4 his3-D1 leu1*-*32 ura4-D18*	This work
Hu1481	h - *hst4Δ*::KanMX6	A-Lien Lu
FY1167	*h-cdc21-HA*::*leu1* + *(mcm4)ura-D18leu1*-*32ade6-M210*	Susan. L. Forsburg
DHP90	*h?-cdc21-HA*::*leu1* + *(mcm4)ura-D18leu1*-*32ade6-M210 hst4Δ*::*his3* +	This work
ENY0670	*h-rad22-YFP*::*kanr leu1*-*32 ura4-D18*	Eishi Noguchi
DHP63	*h-rad22-YFP*::*Kanrleu1*-*32 ura4-D18 hst4Δ*::*his3*+	This work
FY563	*h+cdc10-v50 ura4 leu1*-*32ade6-M210*	Susan. L. Forsburg
DHP91	*h?cdc10-v50 ura4 leu1*-*32ade6-M210hst4Δ*::*his3*+	This work

### Cloning of replication proteins

The *mcl1* gene was amplified and cloned in the BamH1 and Xba1 sites of the pRO314 plasmid. The *mcl1* was also cloned into the Xho1 and Not1 sites of pSLF272 plasmid. The *mcm4* and *sld5* genes were cloned in the Xho1 and Not1 sites of pSLF272 plasmid.

### Cell lines

HeLa cells were cultured in Dulbecco’s Modified Eagle’s Medium (DMEM) supplemented with 10% fetal bovine serum (FBS) and 100 U/mL penicillin and streptomycin in a humidified 5% CO_2_ incubator at 37 °C.

### DNA damage treatment of yeast cells

Cells were grown to OD_600_ = 1. Cultures were subjected to fivefold serial dilution and spotted on YES or EMM plates containing different concentrations of 0.01% MMS or 10 mM HU or 10 µM CPT. The plates were placed in 30 °C incubator for 4–5 days.

### Protein Preparation and Western blotting

Total cell lysates were prepared from 10 mL culture of *S*. *pombe*. Cells were re-suspended in 200 µL of lysis buffer containing 50 mM HEPES, 500 mM NaCl, 5 mM EDTA, 0.1% NP-40, 10% glycerol, 1% protease inhibitor cocktail. Cells were lysed by glass beads using bead beater. Crude extracts were clarified by centrifugation and proteins were estimated through bradford method. Samples were prepared by pre-heating proteins in SDS sample buffer (50 mM Tris pH 7.5, 5 mM EDTA, 5% SDS, 10% glycerol, 0.5% β-mercaptoethanol, 0.05% bromophenol blue) and resolved by SDS-polyacrylamide gel electrophoresis (PAGE) followed by Western blotting to detect specific proteins.

### Live cell Microscopy

Cells expressing Mc1l-GFP and Rad22-YFP were grown to mid-log phase. Cells were pelleted and resuspended in PBS. Live cell miscroscopy was performed on a Confocal microscope (Zeiss, LSM700). Quantification of foci was done by three independent experiments and atleast 300 cells were counted individually. The cell cycle position analysis was performed as earlier^66^. Briefly, Calcofluor was used to stain the septa and hoechst staining was performed to count the nucleus.

### Immunofluorescence

Immunofluorescence was performed as described previously^35^. Briefly, logarithmically growing cells (50 ml) were labelled for 30 min at 25 °C in media containing 150 µg/ml BrdU [Sigma]. Cells were fixed in methanol/paraformaldehyde fixative for 30 min, washed in PBS and then treated with 0.5 mg/ml Zymolyase 20 T and 1 mg/mL lysing enzymes in PEMS for 10 min. After washing in PBS, cells were resuspended in 1 ml of 4 M HCl and incubated for 10 min to denature the DNA. Cells were washed extensively in PBS then blocked in PBS with 10% foetal calf serum for 1 h. Cells were incubated overnight in BrdU antibody [BD Biosciences] at 1:50 in PBS with 10% foetal calf serum and 0.05% Tween-20. Cells were then washed in PBS and incubated with α-mouse-AlexaFluor 488 at 1:500 in PBS with 10% foetal calf serum and 0.05% Tween-20 for 2 h. Cells were washed and resuspended in PBS then put on cover slips previously treated with poly-l-lysine. DNA was detected with 4–6′ diamidino-2-phenylindole (DAPI). Cells were visualized under confocal microscope.

### Measurement of DNA content by flow cytometry

Yeast strains bearing a temperature sensitive *cdc25*-*22* mutation were used for synchronization. The wild type *cdc25*-*22* and *cdc25*-*22 hst4*Δ temperature-sensitive cells transformed with either *hst4* or *mcl1* were arrested in G2 at 36 °C for 4 h and then released at 25 °C for 4 hours and samples were collected every 30 minutes. For G1 arrest, yeast strains bearing a temperature sensitive *cdc10-v50* mutation were used for synchronization. The wild type *cdc10-v50* and *cdc10-v50 hst4*Δ temperature-sensitive cells arrested in G1 at 36 °C for 4 h and then released at 25 °C for 4 hours and samples were collected every 30 minutes. Cells were fixed with 70% ethanol and stained with propidium iodide (PI). Flow cytometry was performed on FACS Scan instrument using Cell Quest software. Histograms were generated using Flow JO (7.6.5).

### Septation Index

The *cdc25*-22 (ROP204) and *cdc25*-*22 hst4Δ* (ROP216) strains were grown at 25 °C to log phase and synchronized in G2 by shifting the cells to 36 °C for 4 h. Cells were then shifted to 25 °C and mitotic progression was determined by 4′,6′-diamidino-2-phenylindole (DAPI) and calcofluor (50 µg/ml) staining. Three hundred cells from each time point were counted and septation index was determined by calculating the percentage of septated cells.

### Slot blot analysis

The slot blot analysis was performed as described previously^67^. Genomic DNA from BrdU incorporated yeast cells was isolated with a commercial kit (zymoresearch). The amount of purified DNA was determined with a nanodrop-spectrophotometer. 500 ng of genomic DNA was made single-stranded by incubating with 10 volumes of 0.4 N NaOH solution for 30 min at room temperature. The denatured DNA was placed on ice and neutralized by an equal volume of 1 M Tris·HCl (pH 6.8). The single-stranded neutralized DNA was diluted in 100 µL water to obtain a series of concentrations of DNA (50 ng, 25 ng and 12.5 ng) and then slot dot blotted onto a nitrocellulose membrane (Amersham) and fixed by ultraviolet cross-linker Stratalinker (Stratagene, La Jolla, CA). To visualize the BrdU signal, the membrane was blocked in 5% non-fat milk and incubated with mouse anti-BrdU monoclonal antibody and the signal was detected by chemiluminiscence. Signal intensities were quantified by densitometric analysis to determine the fold change in BrdU incorporation in the *hst4*Δ mutants compared to wild type.

### RNA isolation and quantitative real-time polymerase chain reaction

RNA was isolated by acid phenol method^68^. Total RNA was used for cDNA synthesis using Superscript III reverse transcriptase (Invitrogen, USA) and the above prepared cDNA was used for RT-qPCR using EvaGreen qPCR Mastermix (GBiosciences, USA). Each sample was run in triplicate and amplification was detected using 7500 Real Time PCR system (Applied Biosystems, USA). Transcripts were normalized to Actin by using −∆∆CT method^69^. Primer sequences used in this assay for Mcl1 are F: AGCTAGTGATGAAACAGCAG and R: GATTCTGCCTCTAAAGAGGC.

### AND-1 rescue experiment

SIRT2 shRNA targeted to 3′ end of SIRT2 mRNA was generated by cloning double stranded oligonucleotides in pGFP-V-RS plasmid, using BamHI and HindIII. The following oligonucleotides were ordered with overhangs: 5′GATCCGCTTATTGGAGACAAATTAAAAACATGTGCTGTCTGTTTTTAATTTGTCTCCAATAAGCTTTTTA 3′complementary strand 5′AGCTTAAAAAGCTTATTGGAGACAAATTAAAAACAGACAGCACATGTTTTTAATTTGTCTCCAATAAGC-3′. HeLa cells were transfected with both vector control and SIRT2 shRNA vector using Lipofectamine 2000 and media change was done after 12 h. Cells were incubated for 48 h before transfecting cells with Flag-SIRT2 for another 24 h.

## Electronic supplementary material

Supplementary data
