# Supplementary material for: Fission Yeast Sirtuin Hst4 Functions in Preserving Genomic Integrity by Regulating Replisome Component Mcl1
Source: Sci Rep. 2018 May 31;8:8496. doi: 10.1038/s41598-018-26476-4 (PMC5981605; doi:10.1038/s41598-018-26476-4)
Supplement: Supplementary file 1 — Supplementary data [file 41598_2018_26476_MOESM1_ESM.pdf]

Supplementary Material

Fission Yeast Sirtuin Hst4 Functions in Preserving Genomic Integrity by Regulating Replisome Component Mcl1

Lahiri Konada[#, a, b], Shalini Arichota[#, a, b], Raghavendra Vadla[a, b], and Devyani Haldar[a]*

[a]Centre for DNA Fingerprinting and Diagnostics, Survey Nos. 728, 729, 730 & 734, Opposite Uppal Water Tank, Beside BSNL T E Building, Uppal, Ranga Reddy District, Hyderabad 500039, India.

[b]Graduate Studies, Manipal University, Manipal, India

# Both authors contributed equally to this work.

*Corresponding author: devyani@cdfd.org.in; Telephone number: 91-40-24749432.

Supplementary Figures

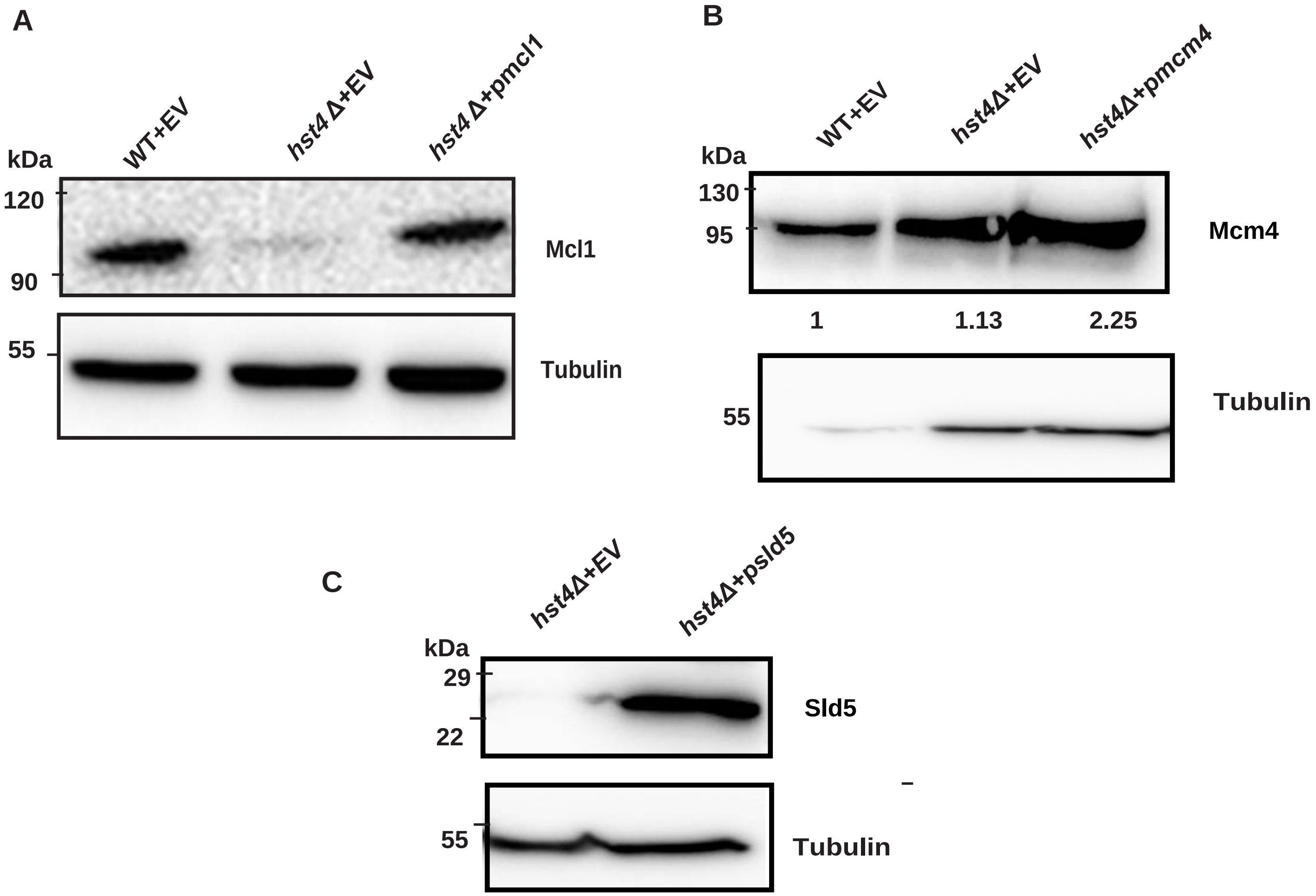

Supplementary Figure S1 - Level of overexpression of Mcl1, Mcm4 and Sld5-

(A) Cell lysates from wild type strain (ROP191) or *hst4* Δ (ROP58) expressing *mcl1* or pRO314 (empty vector) were prepared and Mcl1 levels were analyzed by Western blotting using anti-And-1 antibody. (B) Cell lysates from wild type strain (FY1167) or *hst4*Δ (DHP90) endogenously tagged mcm4-HA expressing *mcm4* or pSLF272 (empty vector) were prepared and Mcm4 levels were analyzed by Western blotting using anti-HA antibody. Numbers showing relative expression normalised to loading control. (C) Cell lysates from wild type strain (ROP191) or *hst4* Δ (ROP58) expressing *sld5* or pSLF272 (empty vector) were prepared and Sld5 levels were analyzed by Western blotting using anti-HA antibody.

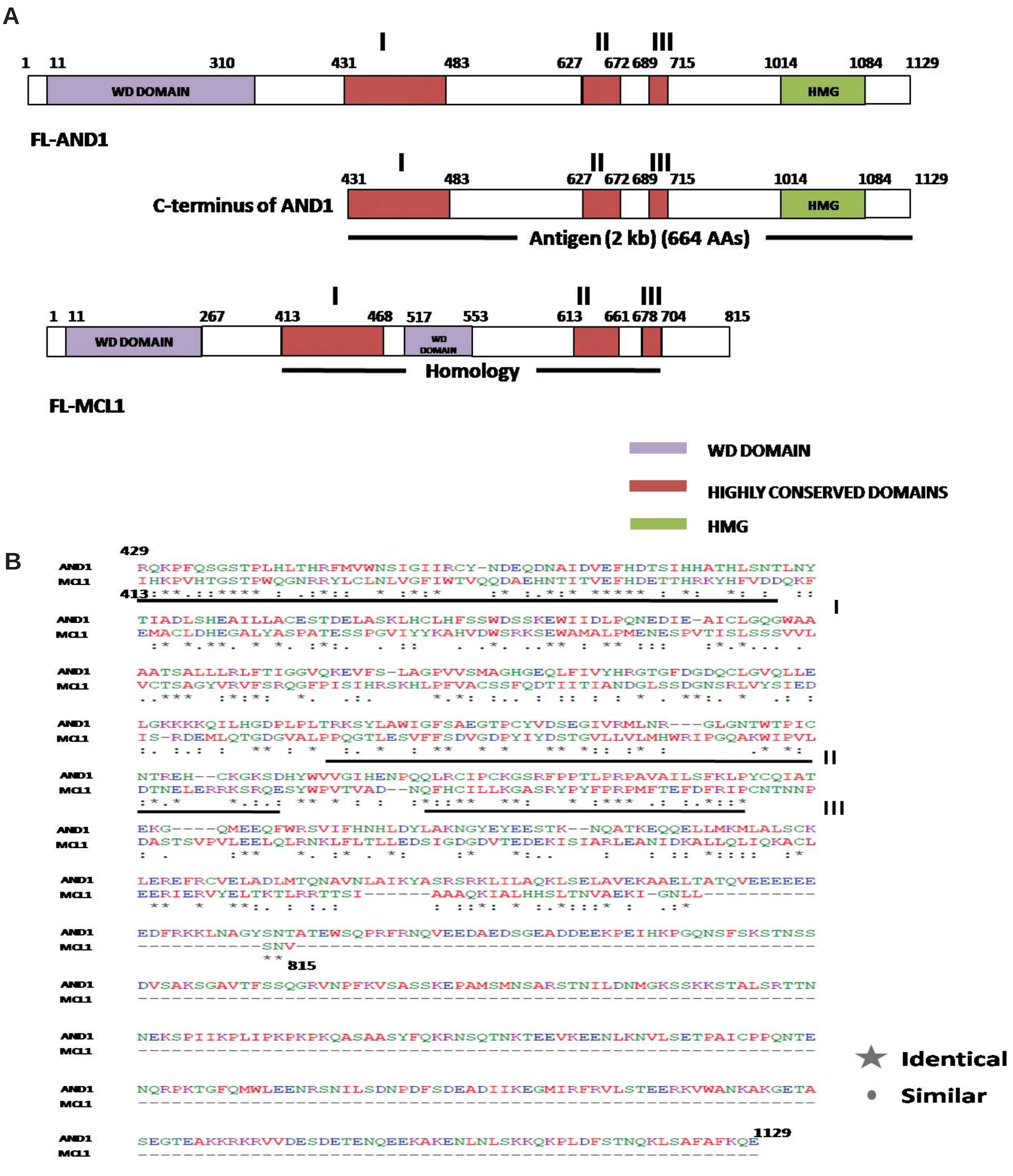

Supplementary Figure S2- Analysis of conserved domains of And-1 and Mcl1 used to generate anti-And-1 antibody.

(A) Cartoon showing conserved domains of full length And-1 and full length Mcl1. The C-terminal region of And-1 was used to raise anti-And-1 antibody. Purple boxes depict WD domains, red boxes depict three sepB domains which is highly conserved amongst the members of And-1/Mcl1 family. Green box depict HMG domain which is unique to And-1. (B) Alignment of C-terminal region of And-1 (429-1129 amino acids) with the C-terminal region of Mcl1 (413-815 amino acids).

References

1. Williams DR, McIntosh JR. mcl1+, the Schizosaccharomyces pombe homologue of CTF4, is important for chromosome replication, cohesion, and segregation. Eukaryot Cell 2002; 1:758-73.

2. Kohler A, Schmidt-Zachmann MS, Franke WW. AND-1, a natural chimeric DNA -binding protein, combines an HMG box with regulatory WD-repeats. J Cell Sci. 1997 ; 110: 1051-62

Uncropped Original Western Blots

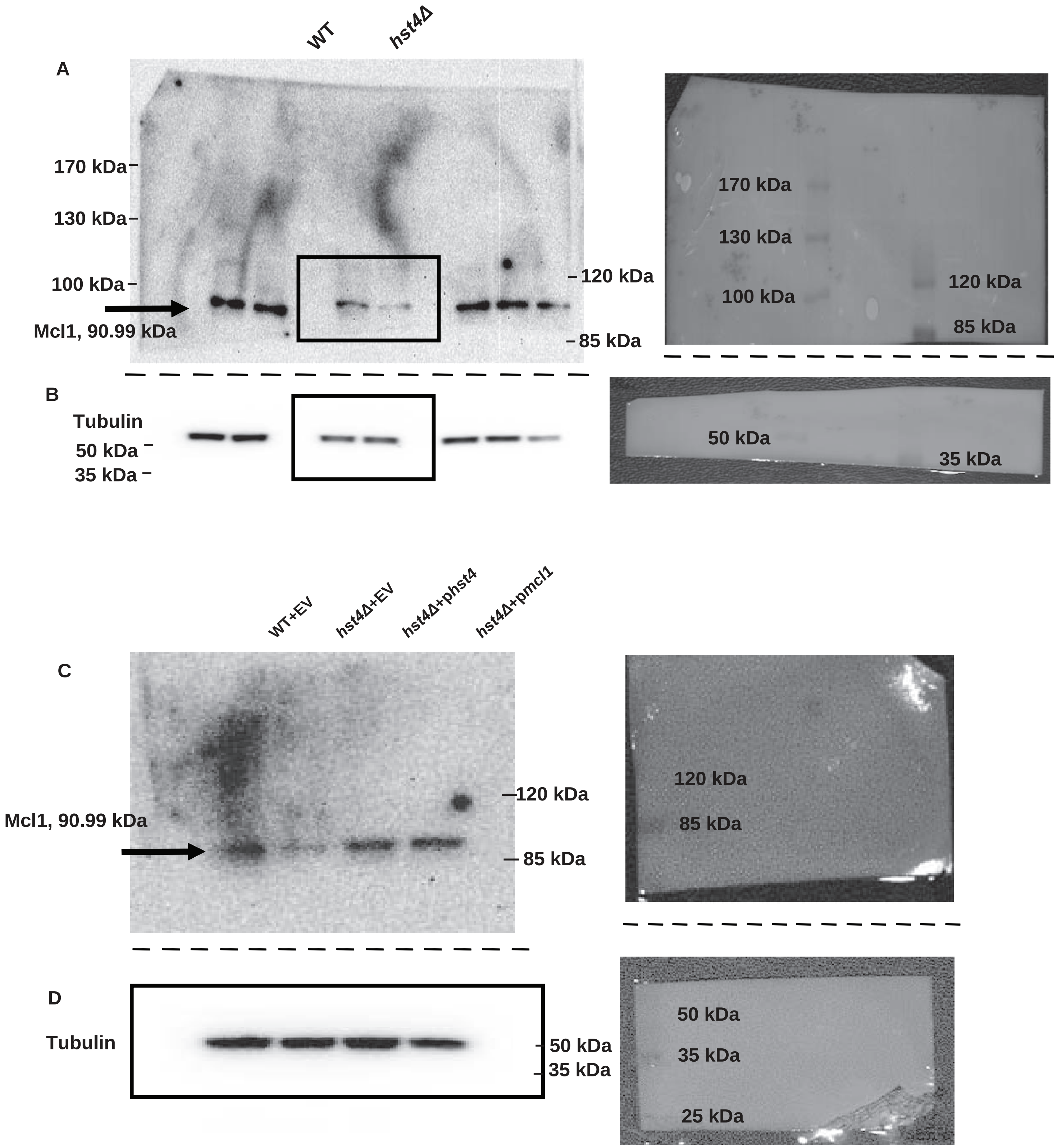

Supplementary Information 1- Uncropped Western Blots for Figure 7B, Wild-type (ROP191) and *hst4*Δ (ROP58) strains were grown asynchronously to mid log phase in YES medium. Whole cell lysates were prepared and the levels of Mcl1 were monitored by Western blotting using anti-And-1 antibody. (A) Highlighted box showing Mcl1 bands used for figure 7B (B) Corresponding loading control for Figure 7B. (C) Uncropped Western blots for Figure 7G , Cell lysates from wild type strain (ROP191) or *hst4*Δ (ROP58) expressing *hst4, mcl1* or pSLF272 (empty vector) were prepared and Mcl1 levels were analyzed by Western blotting using anti-And-1 antibody. (D) Corresponding loading control for Figure 7G. The panels on the right show the corresponding membranes showing molecular weight marker. Dashed lines indicate that the membranes were cut into two.

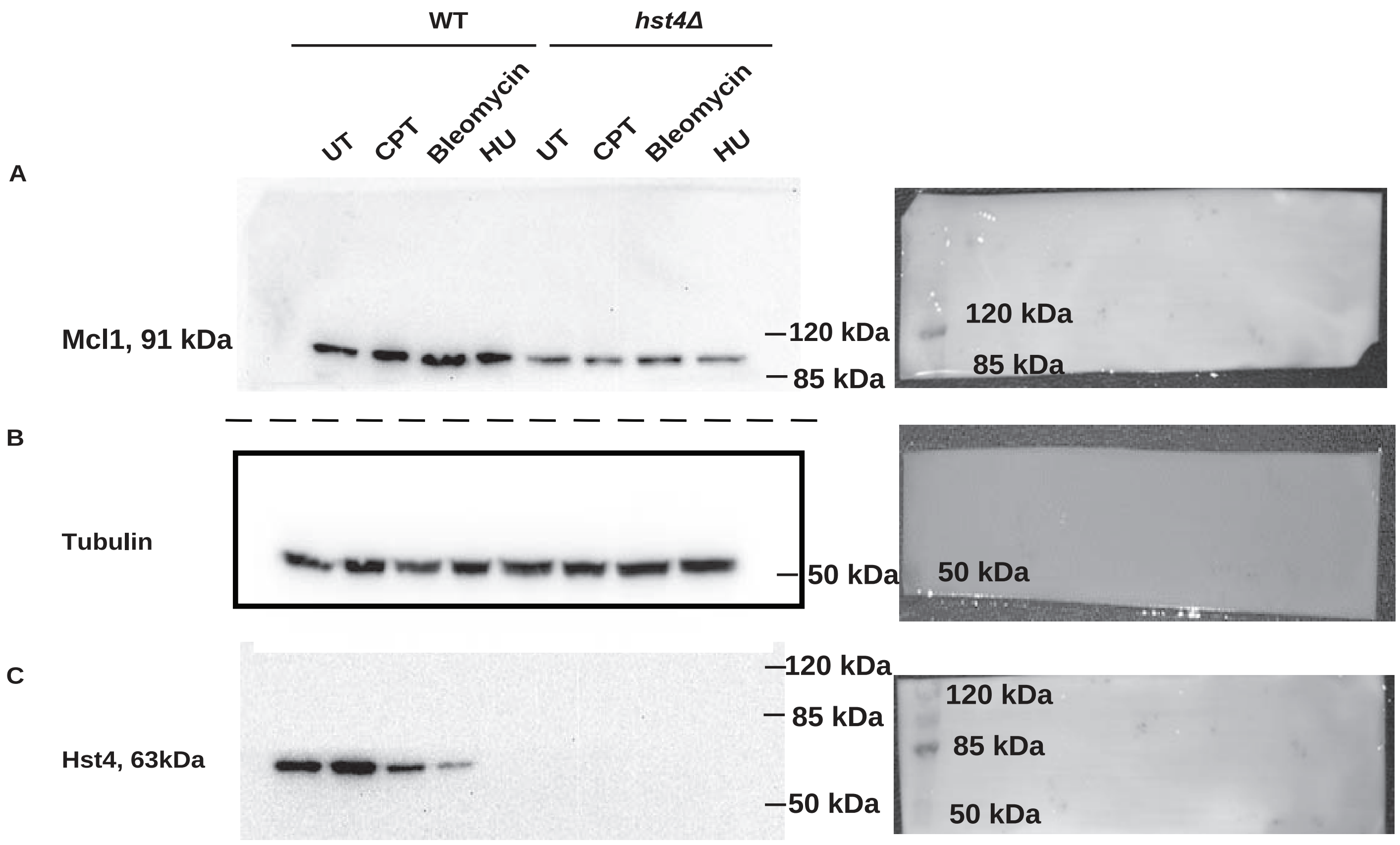

Supplementary Information 2- Uncropped Western Blots for Figure 7K, Assessment of Mcl1 and Hst4 levels by Western blot in WT (ROP191) and *hst4*Δ (ROP58) cells untreated or treated with either CPT or bleomycin or HU (A) Western blot against Mcl1 (B) Corresponding loading control for Figure 7K. (C) Western blot against Hst4 The panels on the right show the corresponding membranes showing molecular weight marker. Dashed lines indicate that the membranes were cut into two.

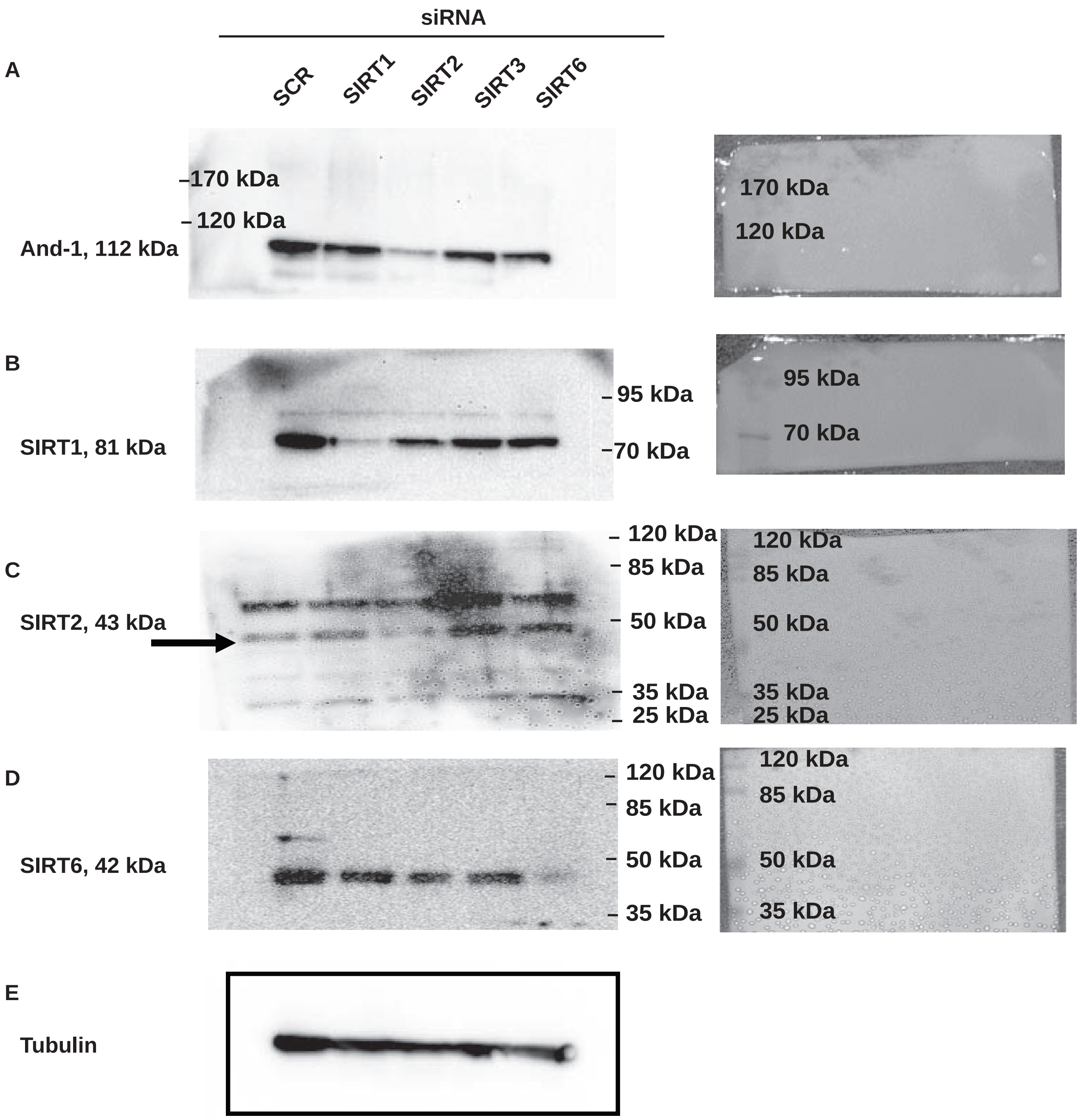

Supplementary Information 3- Uncropped Western Blots for Figure 8A, HeLa cells were transfected with scramble, SIRT1, SIRT2, SIRT3 and SIRT6 siRNA. At 48 h post transfection, whole cell extracts were prepared and detected by Western blot. (A) Western Blot against And-1 (B) Western Blot against Sirtuin 1 (C) Western Blot against Sirtuin 2 (D) Western Blot against Sirtuin 6 (E) Corresponding loading control for Figure 8A. The panel on the right show corresponding membranes showing molecular weight markers.

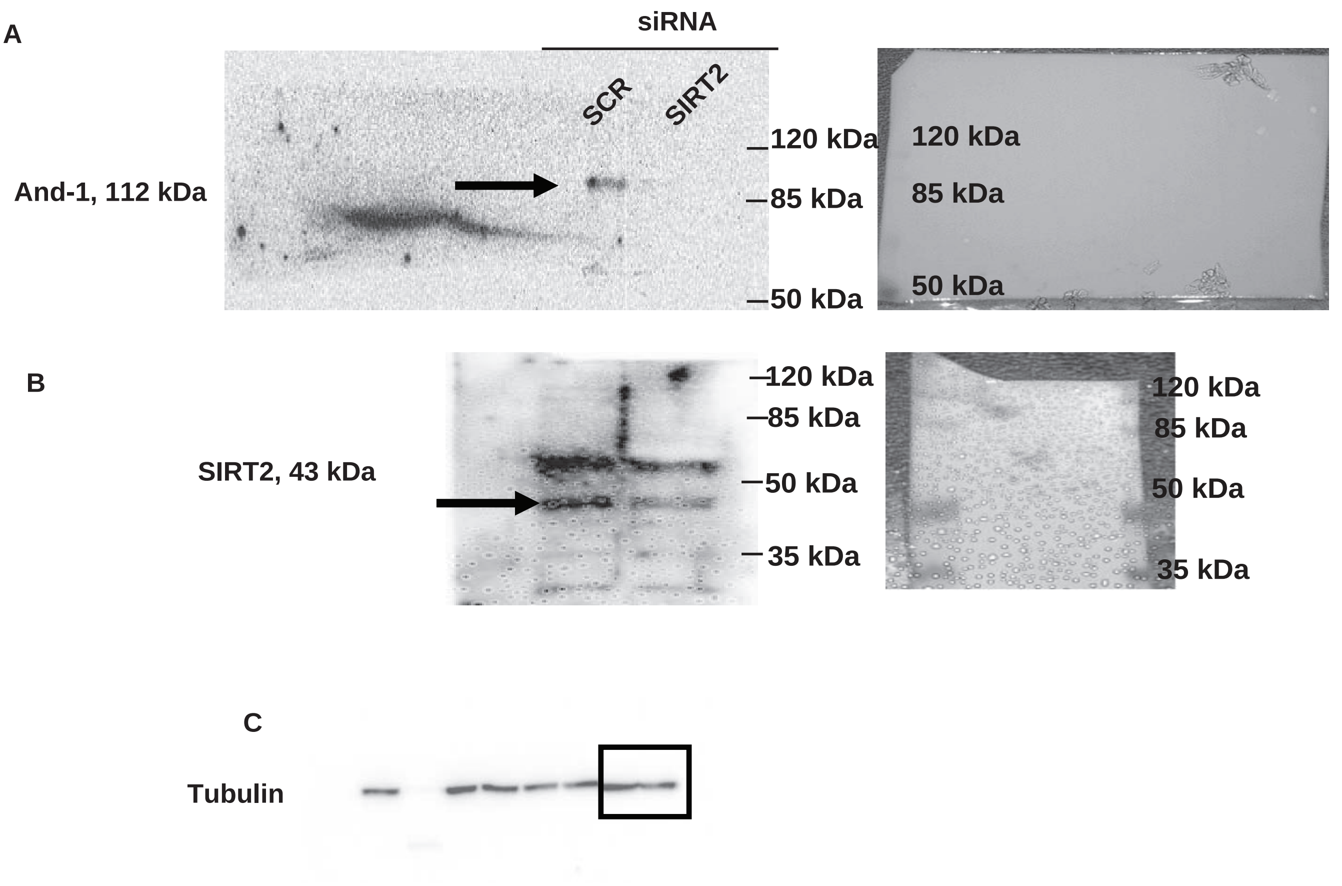

Supplementary Information 4- Uncropped Western Blots for Figure 8B, HeLa cells were transfected with scramble and SIRT2 siRNA. At 48 h post transfection, whole cell extracts were prepared and detected by Western blot. (A) Western Blot against And-1 (B) Western Blot against Sirtuin 2 (C) Highlighted box showing corresponding loading control for Figure 8B. The panels on the right show the corresponding membranes showing molecular weight marker.

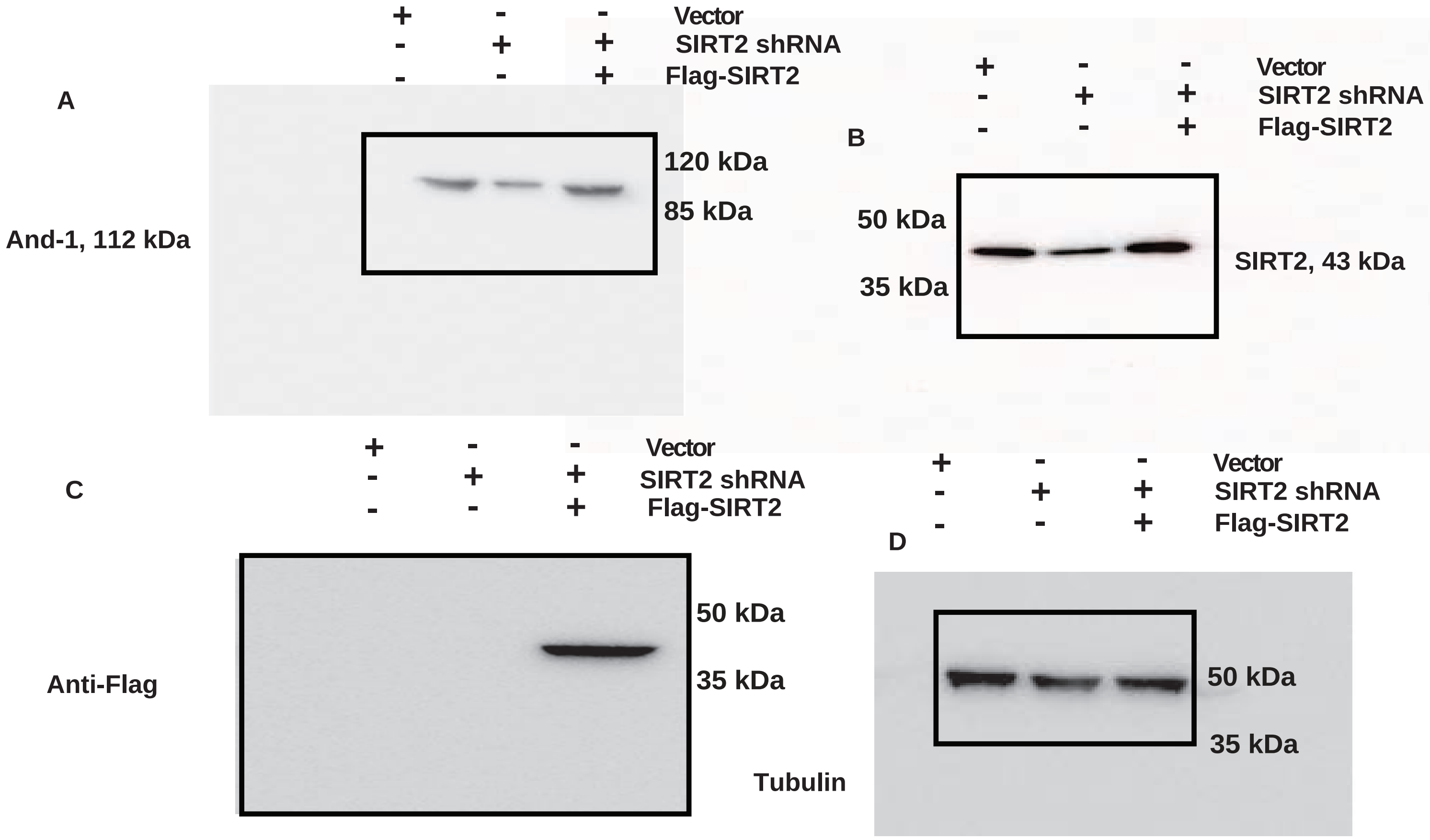

Supplementary Information 5- Uncropped Western Blots for Figure 8C, HeLa cells were transfected with SIRT2 shRNA targeted to 3'end of endogenous mRNA for 48 h and then transfected with Flag-SIRT2 for 24 h. (A) Western Blot against And-1 (B) Western Blot against Sirtuin 2 (C) Western Blot against overexpressed Sirtuin 2 using Anti-Flag antibody (D) Corresponding loading control for Figure 8C.

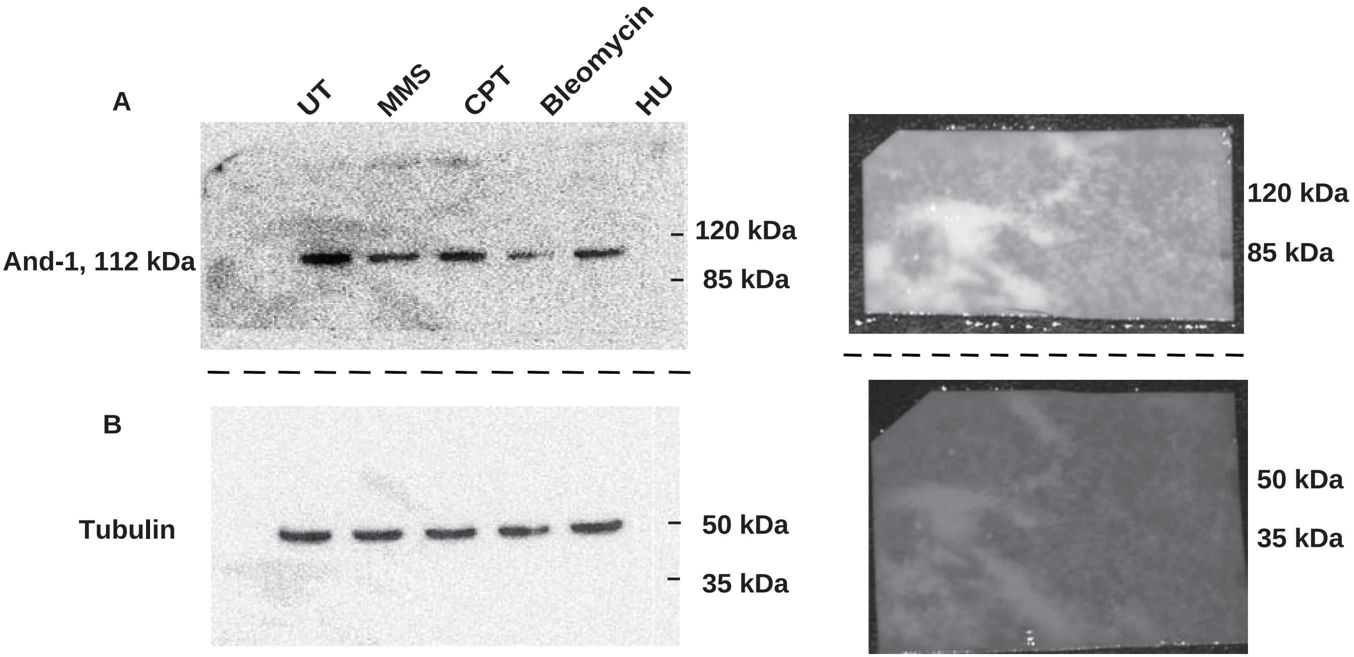

Supplementary Information 6- Uncropped Western Blots for Figure 8E, And-1 expression in HeLa cells treated with indicated damaging agents. (A) Western Blot against And-1 (B) Corresponding loading control for Figure 8E. The panels on the right show the corresponding membranes showing molecular weight marker. Dashed lines show that the membrane was cut into two.

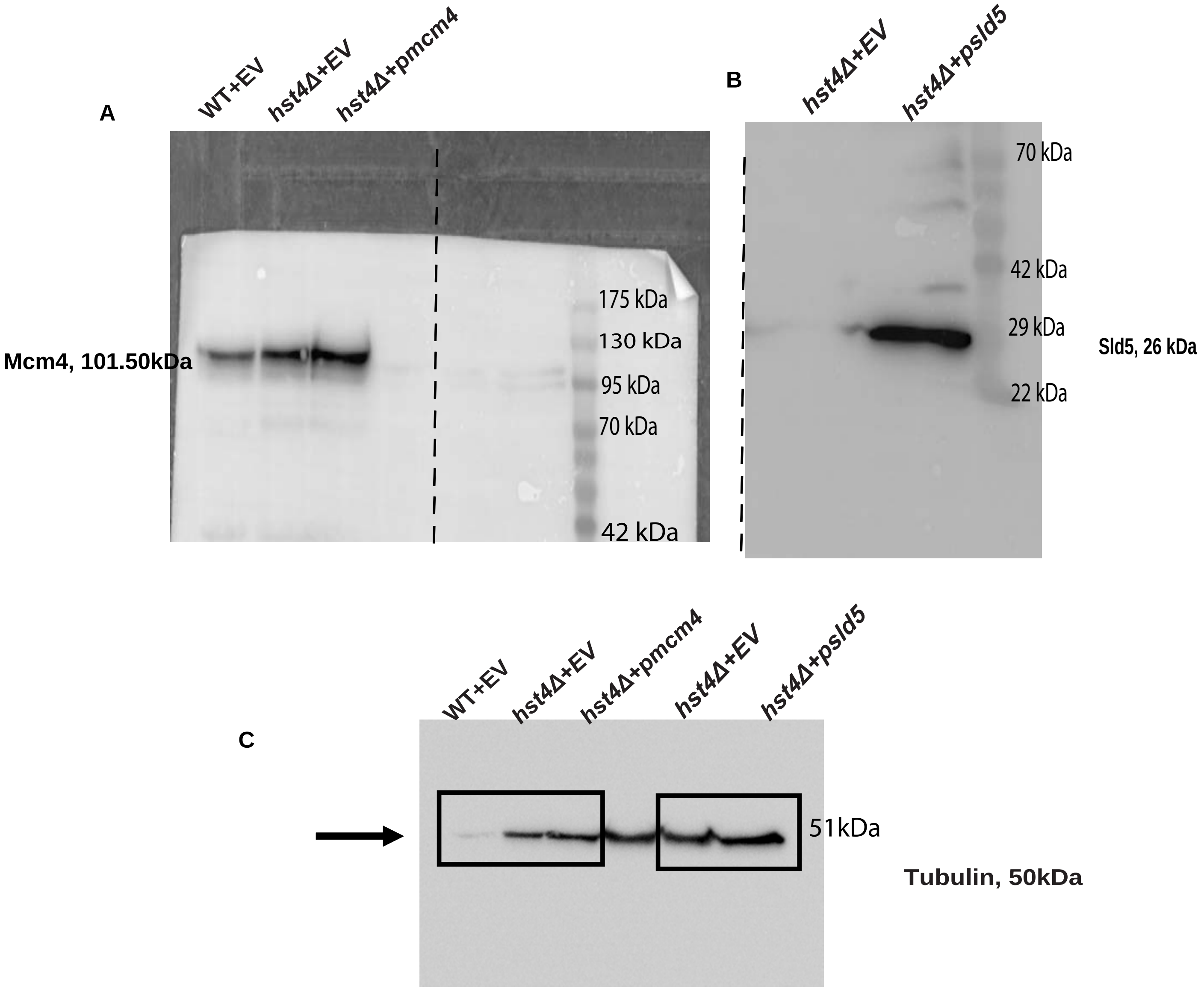

Supplementary Information 7- Uncropped Western Blots for Supplementary figure S1B and C, (A) Western Blot against Mcm4 (B) Western Blot against Sld5 (C) Corresponding loading control. Dashed lines indiacte samples run on the same blot however masked while developing due to background bands on overexposure.

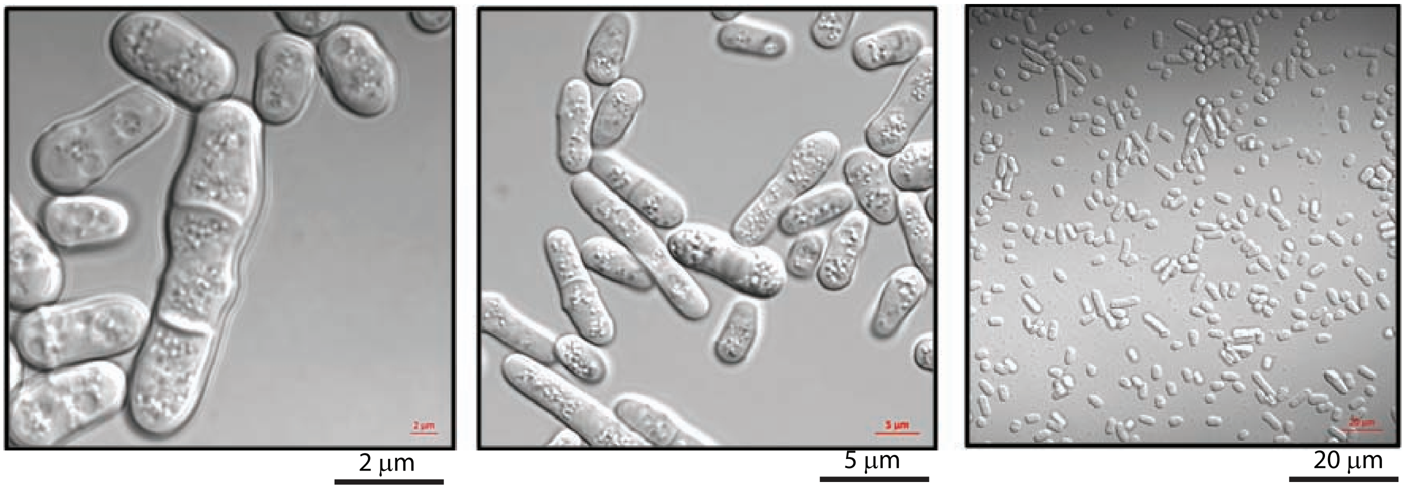

Supplementary Information 8- DIC images of *S pombe* cells viewed under different scales.
